# MyD88/CD40 signaling retains CAR T cells in a less differentiated state

**DOI:** 10.1172/jci.insight.136093

**Published:** 2020-11-05

**Authors:** Brooke Prinzing, Patrick Schreiner, Matthew Bell, Yiping Fan, Giedre Krenciute, Stephen Gottschalk

**Affiliations:** 1 Department of Bone Marrow Transplantation and Cellular Therapy, St. Jude Children’s Research Hospital, Memphis, Tennessee, USA.; 2 Graduate School of Biomedical Sciences, Baylor College of Medicine, Houston, Texas, USA.; 3 Center for Applied Bioinformatics and; 4 Graduate School of Biomedical Sciences, St. Jude Children’s Research Hospital, Memphis, Tennessee, USA.

**Keywords:** Immunology, Therapeutics, Cancer immunotherapy, Immunotherapy, T cells

## Abstract

Chimeric antigen receptor (CAR) T cell therapy for solid tumors has shown limited efficacy in early-phase clinical studies. The majority of CARs encode CD28 and/or 41BB costimulatory endodomains, and we explored whether MyD88 and CD40 (MC) costimulatory endodomains in CARs could improve their antitumor activity. We generated CD28-, 41BB-, and MC-CAR T cells and demonstrated that MC-CAR T cells have greater proliferative capacity and antitumor activity in repeat stimulation assays and in tumor models in vivo. Transcriptomic analysis revealed that MC-CAR T cells expressed higher levels of *MYB* and *FOXM1*, key cell cycle regulators, and were activated at baseline. After stimulation, MC-CAR T cells remained in a less differentiated state than CD28- and 41BB-CAR T cells as judged by low levels of transcription factor TBET and B lymphocyte induced maturation protein 1 expression and lower cytolytic activity in comparison with CD28- and 41BB-CAR T cells. Thus, including MyD88 and CD40 signaling domains in CARs may improve current CAR T cell therapy approaches for solid tumors.

## Abstract 2

Including MyD88 and CD40 in the signaling domain of CARs limits terminal effector differentiation of CAR T cells, resulting in enhanced antitumor activity.

## Graphical abstract

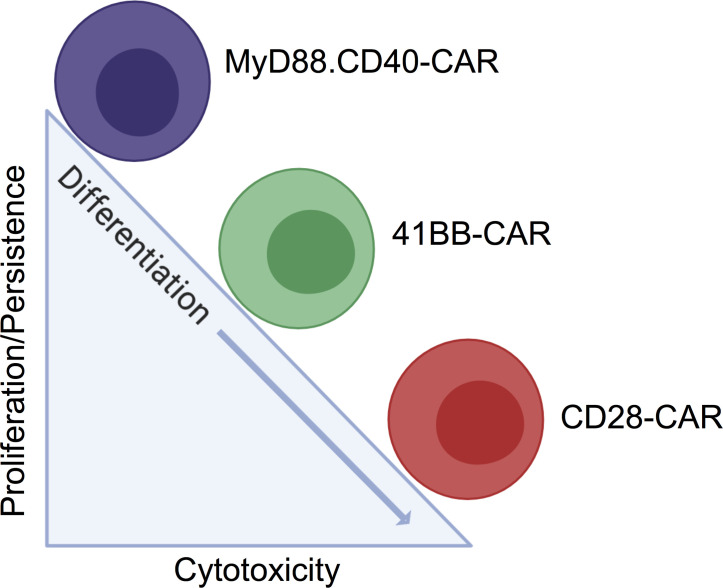

## Introduction

Immunotherapy with T cells expressing chimeric antigen receptors (CAR T cells) had remarkable success for CD19^+^ hematological malignancies, leading to their FDA approval (1–3). However, the adoptive transfer of CAR T cells for solid tumors and brain tumors has had limited clinical success so far (4–6). This lack of efficacy is most likely multifactorial, including heterogenous antigen expression, limited homing of infused T cells to tumor sites, and reduced CAR T cell function within the tumor microenvironment (7).

Several approaches are currently being pursued to improve the effector function of CAR T cells, including transgenic expression of cytokines or knocking out negative regulators that inhibit T cell function (8–11). We and others have previously shown that expression of an inducible costimulatory molecule that consists of 2 FKBP12_v36_ binding domains, MyD88, and CD40 (iMC) significantly enhances the effector function of T cells expressing a CAR with a CD3 ζ endodomain (ζ-CAR) (12, 13). More recently, investigators showed that constitutive expression of MyD88 and CD40 (cMC) improved the effector function of T cells expressing CARs with a ζ-signaling domain, while incorporating MyD88 and CD40 signaling domain into the signaling domain of a CD19-specific CAR reduced its antitumor activity (14). MyD88, the canonical adaptor molecule for TLR and IL-1 receptor family signaling (15), and CD40, a known T cell costimulatory molecule with a role in memory formation (16, 17), are both well-studied molecules. However, mechanistic studies with iMC and cMC ζ-CAR T cells have been limited, showing decreased PD-1 expression after stimulation of iMC and cMC CAR T cells; activation of NF-κB, TNF, and antiapoptotic pathway signaling after iMC activation; and baseline cytokine expression of cMC CAR T cells (12–14).

To define the signaling pathways that differ between CARs with CD28.ζ (CD28-CAR), 41BB.ζ (41BB-CAR), and MyD88.CD40.ζ (MC-CAR), we generated CARs specific for EphA2, a tumor-associated antigen broadly expressed in brain and solid tumors (18, 19). We demonstrate that MC-CAR T cells have superior effector function in vitro and in vivo in comparison with CD28- and 41BB-CAR T cells. Transcriptomic analysis revealed significant similarities between 41BB- and MC-CAR T cells. However, after stimulation MC-CAR T cells retained a less differentiated phenotype as judged by lower levels of transcription factors associated with terminally differentiated T cells such as TBET and B lymphocyte induced maturation protein 1 (BLIMP1) and effector molecules such as granzyme B.

## Results

### Generation of T cells expressing EphA2-CARs with an MyD88.CD40.ζ endodomain.

**Figure 1:**
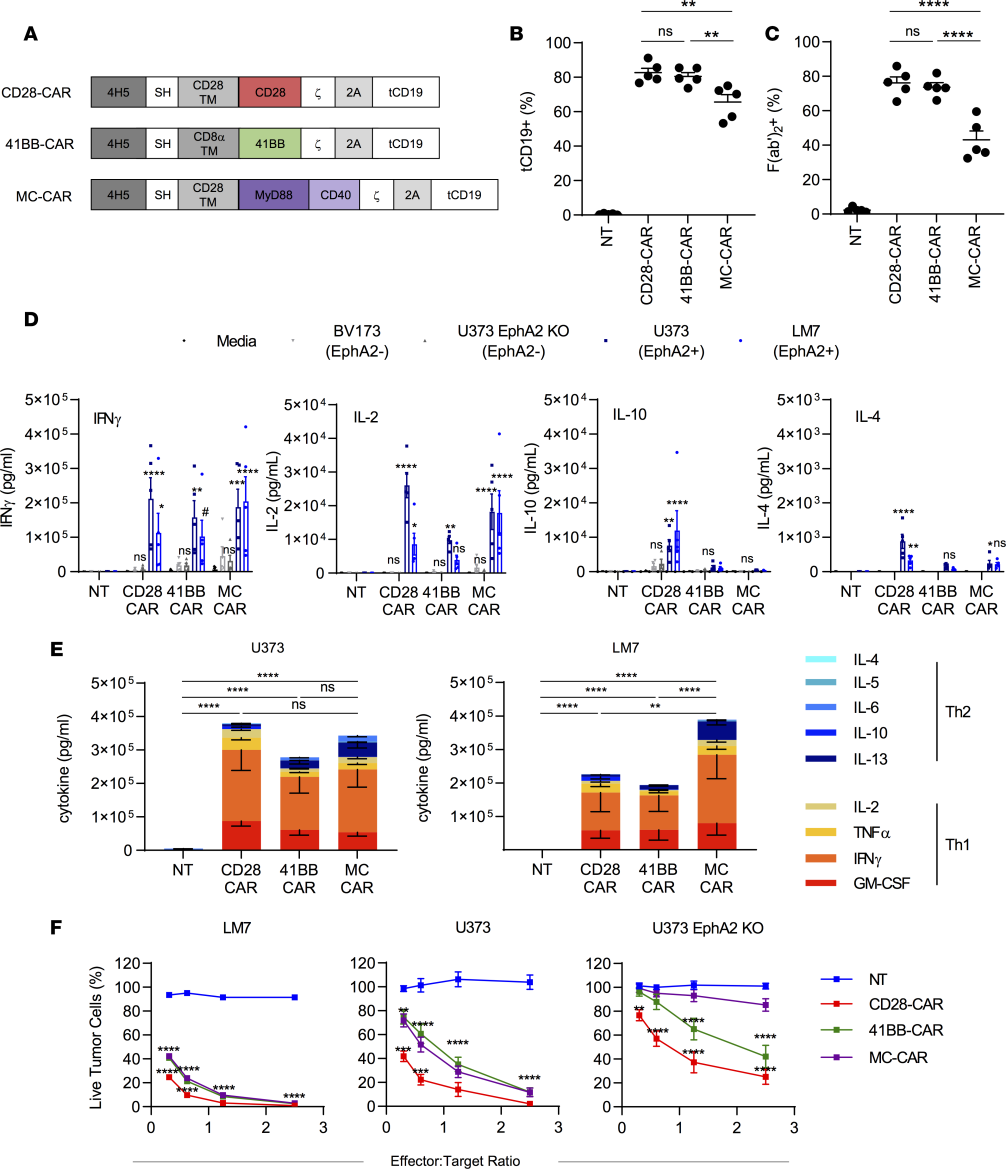
Generation and functional characteristics of CAR T cells. (**A**) Scheme of EphA2-CAR constructs. (**B**) Summary plot of %tCD19^+^ T cells (*n* = 5, mean ± SEM, 1-way ANOVA with Tukey’s test for multiple comparisons). (**C**) Summary plot of %F(ab′)_2_-positive T cells (*n* = 5, mean ± SEM, 1-way ANOVA with Tukey’s test for multiple comparisons). (**D**) CAR T cell production of Th1 (IFN-γ and IL-2) and Th2 (IL-4 and IL-10) cytokines after 24-hour coculture at a 2:1 ratio against EphA2-positive (LM7, U373 WT) and EphA2-negative (BV173, U373 EphA2 KO) cell lines or in media alone (*n* = 5, mean ± SEM, 2-way ANOVA with Dunnett’s test for multiple comparisons, all statistical analysis is in comparison with NT cells). Dot colors: black, media; light gray, BV173 (EphA2 negative); dark gray, U373 EphA2 KO (EphA2 negative); dark blue: U373 (EphA2 positive); light blue, LM7 (EphA2 positive). (**E**) Summary plots of Th1 and Th2 cytokine production against EphA2-positive cell lines U373 and LM7 (*n* = 5, mean ± SEM, values were log transformed before 2-way ANOVA with Tukey’s test for multiple comparisons). (**F**) CAR T cells were incubated with increasing amounts of tumor cells for 24 hours, and the remaining live tumor cells were quantified with an MTS assay (2-way ANOVA with Tukey’s test for multiple comparisons, mean ± SEM, LM7: *n* = 4, U373 WT and U373 EphA2 KO: *n* = 9). For LM7 and U373, asterisks refer to statistical comparison of MC-CAR with NT and CD28-CAR with MC-CAR. For U373 EphA2 KO, asterisks refer to statistical comparison of 41BB-CAR with NT and CD28-CAR with NT. ^#^*P* < 0.1; *P < 0.05; **P < 0.01; ***P < 0.001; ****P < 0.0001.

We generated a retroviral vector encoding an EphA2-CAR based on the EphA2-specific scFv 4H5 (20) with a CD28 transmembrane domain and a MyD88.CD40.ζ endodomain (MC-CAR). In addition, the vector encoded a 2A sequence and truncated CD19 (tCD19; Figure 1A). As controls we used 2 previously generated retroviral vectors encoding EphA2-CARs with CD28.ζ (CD28-CAR) or 41BB.ζ (41BB-CAR) endodomains, a 2A sequence, and tCD19 (Figure 1A) (21). CAR T cells were generated by retroviral transduction of CD3/CD28-activated PBMCs from healthy donors. While more than 50% of each CAR T cell population was transduced, the median transduction efficiency was significantly lower for MC-CAR T cells as judged by tCD19 or CAR expression (Figure 1, B and C; Supplemental Figure 1, A and B; supplemental material available online with this article; https://doi.org/10.1172/jci.insight.136093DS1). Generated CAR T cells contained a mixture of CD4^+^ and CD8^+^ T cells with no significant difference between the 3 CAR constructs and nontransduced (NT) T cells (Supplemental Figure 1C). MC-CAR T cells contained significantly more (*P* < 0.05) central memory (CCR7^+^CD45RA^–^) T cells in comparison with CD28-CAR and NT T cells. This was mirrored by a decrease of terminally differentiated effector memory (CCR7^–^CD45RA^–^) T cells, which only reached significance for CD28-CAR T cells (Supplemental Figure 1D).

### MC-CAR T cells recognize and kill target cells in an antigen-dependent manner.

To initially assess the effector function of MC-CAR T cells, we performed coculture assays to determine cytokine production and cytotoxicity. NT, CD28-CAR, and 41BB-CAR T cells served as controls. We used EphA2-positive (U373, LM7) and EphA2-negative (BV173) tumor cell lines as target cells (Supplemental Figure 2). In addition, we generated EphA2-negative U373 cells by knocking out EphA2 (U373 EphA2 KO) using CRISPR/Cas9 gene editing (Supplemental Figure 2). CD28-, 41BB-, and MC-CAR T cells secreted significant amounts of Th1 (IFN-γ, IL-2, TNF-α, and GM-CSF) and Th2 cytokines (IL-4, IL-5, IL-6, IL-10, and IL-13) after stimulation with EphA2-positive target cells in comparison with NT T cells (Figure 1, D and E; Supplemental Figure 3). Cytokine production was EphA2 dependent because EphA2-negative target cells induced minimal cytokine production. Although CD28-, 41BB-, and MC-CAR T cells produced Th1 and Th2 cytokines, production of Th1 cytokines was significantly greater (*P* < 0.05) for all CAR constructs after stimulation with LM7 and U373 (Figure 1E).

CD28-, 41BB-, and MC-CAR T cells killed LM7 and U373 cells in a 24-hour MTS cytotoxicity assay in contrast with NT T cells (Figure 1F). Although CD28-CAR T cells had significantly (*P* < 0.001) greater cytolytic activity than MC-CAR T cells at low effector-to-target (E/T) ratios (<1:1), we also observed significant (*P* < 0.01) nonspecific killing of U373 EphA2 KO. 41BB-CAR T cells had similar antitumor activity in comparison with MC-CAR T cells but also killed U373 EphA2 KO, especially at high E/T ratios (Figure 1F).

### MC-CAR T cells exhibit superior performance in repeat stimulation assay.

**Figure 2:**
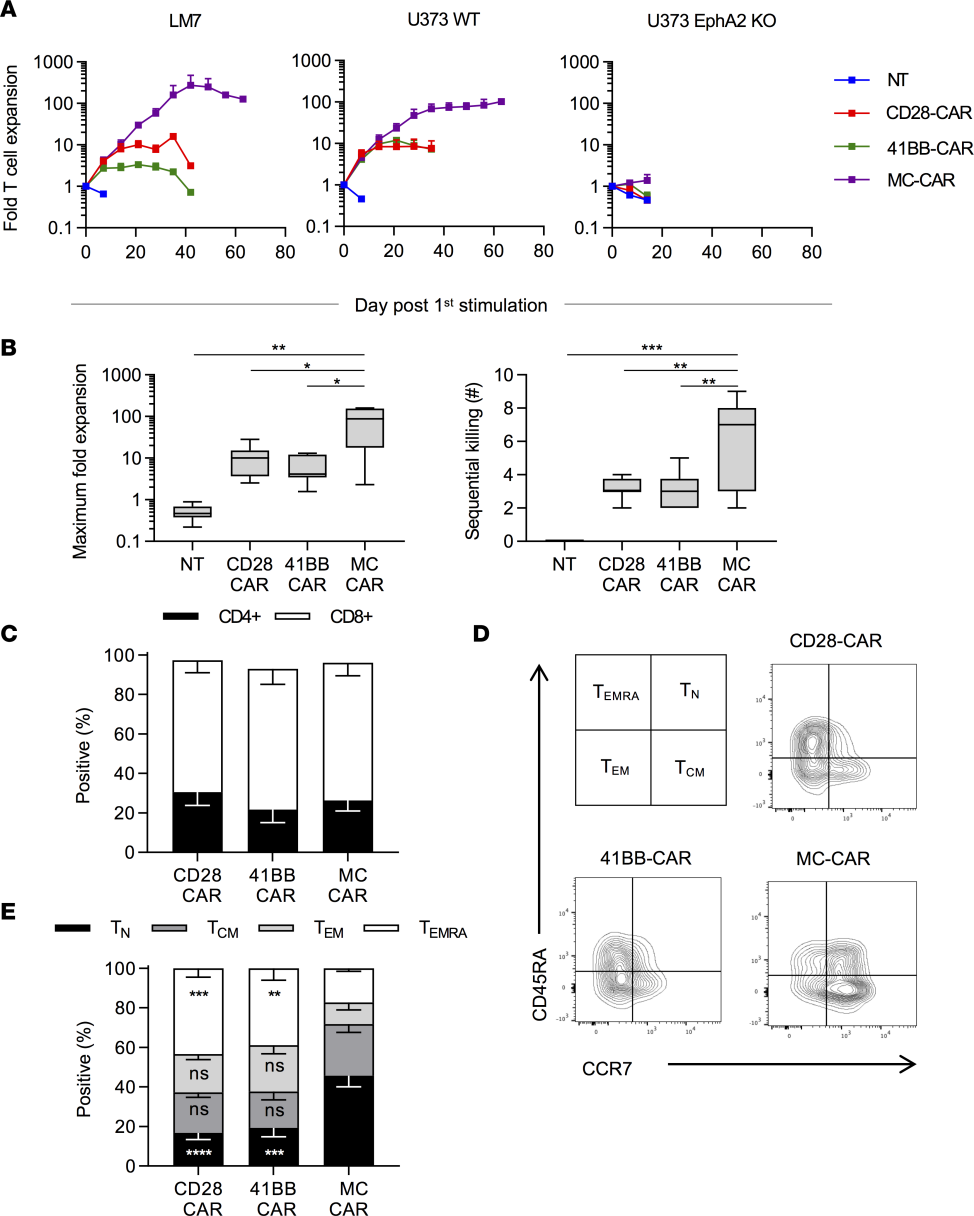
MC-CAR T cells outperform CD28 and 41BB in repeat stimulation assay. T cells were cocultured with tumor cells at a 2:1 ratio with weekly restimulation against fresh tumor cells until they lost their effector function and no longer killed all the tumor cells. (**A**) Average expansion of CAR T cells against EphA2-positive (U373 and LM7) and U373 EphA2 KO cell line (mean ± SEM, LM7: *n* = 4; U373: *n* = 8 [NT, CD28, MC], *n* = 4 [41BB]; U373 KO: *n* = 8 [NT, CD28, MC], *n* = 6 [41BB]). (**B**) Summary of the maximum expansion CAR T cells from individual donors achieved against EphA2-positive tumor cells and the maximum number of times CAR T cells were able to kill fresh EphA2-positive tumor cells (*n* = 12 [NT, CD28, and MC], *n* = 8 [41BB]; median and quartiles, 1-way ANOVA with Tukey’s test for multiple comparisons). T cells were phenotyped 7 days after stimulation with U373. (**C**) Summary plot of CD4/CD8 composition after stimulation with U373 (*n* = 3, mean ± SEM). (**D**) Scheme for phenotyping T cells and representative flow cytometry plots of CCR7 and CD45RA expression on CAR T cells after stimulation with U373. (**E**) Summary plot of T cell phenotype after stimulation with U373 (*n* = 4, mean ± SEM, 2-way ANOVA with Tukey’s test for multiple comparisons). All statistical tests were performed in comparison with MC-CAR T cells (*P < 0.05; **P < 0.01; ***P < 0.001; ****P < 0.0001).

Because we only observed minor differences between all 3 CAR T cell populations after a single exposure to EphA2-positive target cells, we performed repeat stimulation assays. T cells were cocultured with LM7, U373, or U373 EphA2 KO at an E/T ratio of 2:1. Every 7 days, T cells were counted and cocultured again with tumor cells at the same E/T ratio until they no longer killed tumor cells. After the first stimulation with LM7 or U373, there were no differences in expansion between MC-, CD28- or 41BB-CAR T cells (Figure 2A). However, with repeat stimulations, the median fold expansion of MC-CAR T cells was 87-fold, which was significantly greater (*P* < 0.05) than for CD28- (10-fold) or 41BB-CAR T cells (4-fold) (Figure 2, A and B). MC-CAR T cells also had a significantly greater sequential killing capacity (*P* < 0.01) than CD28- or 41BB-CAR T cells (Figure 2B). Improved effector function of MC-CAR T cells was strictly antigen dependent because no difference between CAR T cell populations was observed in the presence of U373 EphA2 KO (Figure 2A). After the first stimulation, phenotypic analysis revealed a preferential expansion of CD8^+^ T cells (Figure 2C vs. Supplemental Figure 1C). MC-CAR T cells had a significantly greater percentage (*P* < 0.001) of naive-like T cells (CCR7^+^CD45RA^+^) and a lower percentage (*P* < 0.01) of terminally differentiated effector T cells (CCR7^–^CD45RA^+^) in comparison with CD28- or 41BB-CAR T cells (Figure 2, D and E). However, this difference in phenotype was not sustained with the second stimulation, and the predominant phenotype of MC-CAR T cells at later stimulations was effector memory (CCR7^–^CD45RA^–^) (Supplemental Figure 4A). We also performed flow cytometry for lymphocyte activating 3 (LAG3), programmed cell death 1 (PD-1), and T-cell immunoglobulin and mucin domain-containing protein 3 (TIM3) 7 days after the first and third stimulations with LM7 cells using T cells that expressed an EphA2-CAR without a signaling domain (Delta-CAR) (21) as a control. Although CD28-, 41BB-, and MC-CAR T cells expressed higher levels of LAG3, PD-1, and TIM3 after the first stimulation in comparison with Delta-CAR T cells, the only sustained difference we observed between T cells expressing functional CARs was significantly lower PD-1 expression on MC-CAR T cells 7 days after the third stimulation (Supplemental Figure 4B). We also examined expression of the activation markers CD69, 41BB, and CD25 at 24 and 72 hours after stimulation with LM7 tumor cells (Supplemental Figure 5). MC-CAR T cells expressed similar levels of all 3 activation markers as CD28-CAR T cells. However, MC-CAR T cells expressed significantly more CD69 (*P* < 0.01) and 41BB (*P* < 0.05) than 41BB-CAR T cells 24 hours after stimulation.

### MC-CAR T cells exhibit lower susceptibility to activation-induced cell death.

We next sought to discern whether the enhanced expansion of MC-CAR T cells was due to lower susceptibility to activation-induced cell death (AICD) or more proliferation. T cells were stimulated with recombinant human EphA2 (rhEphA2) protein for 24 hours before staining with the dead cell dye LIVE/DEAD Aqua (LDA) and annexin V (Supplemental Figure 6, A and B). MC-CAR T cells underwent some AICD as compared with the nonsignaling Delta-CAR T cells (Supplemental Figure 6, A and B). However, the percentage of live (annexin V^–^LDA^–^) MC-CAR T cells poststimulation was significantly greater than in the CD28-CAR (*P* < 0.0001) or 41BB-CAR (*P* < 0.01) T cells. Compared with MC-CAR T cells, the CD28-CAR T cells had a significantly greater (*P* < 0.0001) percentage of preapoptotic (annexin V^+^LDA^–^) cells whereas the 41BB-CAR T cells had a significantly larger (*P* < 0.05) proportion of dead (annexin V^+^LDA^+^) cells. This correlated with a significant higher expression of the antiapoptotic protein Bcl-2 in MC-CAR T cells than CD28- (*P* < 0.05) or 41BB-CAR (*P* < 0.01) T cells (Supplemental Figure 6, C and D). Additionally, when stimulated with LM7 tumor cells, MC-CAR T cells expressed significantly higher levels of the antiapoptotic protein Bcl-xL at both day 3 (*P* < 0.01) and day 7 (*P* < 0.001) poststimulation (Supplemental Figure 6, E and F). In order to assess proliferation, CAR T cells were stimulated with LM7 tumor cells, and expression of the proliferative marker Ki-67 was assessed at day 3 and day 7 poststimulation (Supplemental Figure 6, G and H). Although there was no difference on day 3, by day 7, MC-CAR T cells expressed significantly higher (*P* < 0.01) levels of Ki-67, indicative of sustained proliferation poststimulation.

### Improved MC-CAR T cell performance does not depend on low levels of CAR expression.

Up to now our comparison was done with CAR T cell populations that differed in the level of CAR expression (Figure 1, B and C; and Supplemental Figure 1, A and B). To exclude this as a confounding factor, we generated low-MFI CD28- and 41BB-CAR T cells by placing tCD19 and an internal ribosome entry site (IRES) upstream of the CD28- or 41BB-CAR (Supplemental Figure 7A; IRES CD28-CAR, IRES 41BB-CAR). IRES CD28- or IRES 41BB-CARs were expressed on the T cell surface at similar levels to the MC-CAR as judged by flow cytometry (Supplemental Figure 7, B and C). In a 24-hour cytotoxicity assay against LM7, IRES CD28-CAR T cells were significantly (*P* < 0.0001) more cytotoxic than MC-CAR T cells at low E/T ratios (<1:1), whereas the IRES 41BB-CAR T cells exhibited similar cytotoxicity to the MC-CAR T cells (Supplemental Figure 8A). In our repeat stimulation assay, MC-CAR T cells expanded significantly more (*P* < 0.05) than IRES-CD28 or IRES-41BB CAR T cells and also retained their ability to kill fresh tumor cells for more stimulations (Supplemental Figure 8B). Phenotypic analysis of CAR T cells 7 days after stimulation revealed a significant (*P* < 0.0001) difference between both IRES CAR T cell populations and MC-CAR T cells. Whereas the IRES CARs both had a predominantly effector memory (CCR7^–^CD45RA^–^) phenotype, MC-CAR T cells exhibited a predominantly central memory (CCR7^+^CD45RA^–^) phenotype (Supplemental Figure 8, C and D). We performed the same assays with IRES CAR T cells to interrogate AICD and T cell proliferation poststimulation (Supplemental Figure 8, E–J) and found the same significant differences we had demonstrated when comparing CD28-, 41BB-, and MC-CAR T cells. Thus, the improved performance of MC-CAR T cells cannot be explained by different levels of CAR expression on the cell surface of T cells.

### MC-CAR T cells have greater antitumor activity than CD28- and 41BB-CAR T cells in vivo.

**Figure 3:**
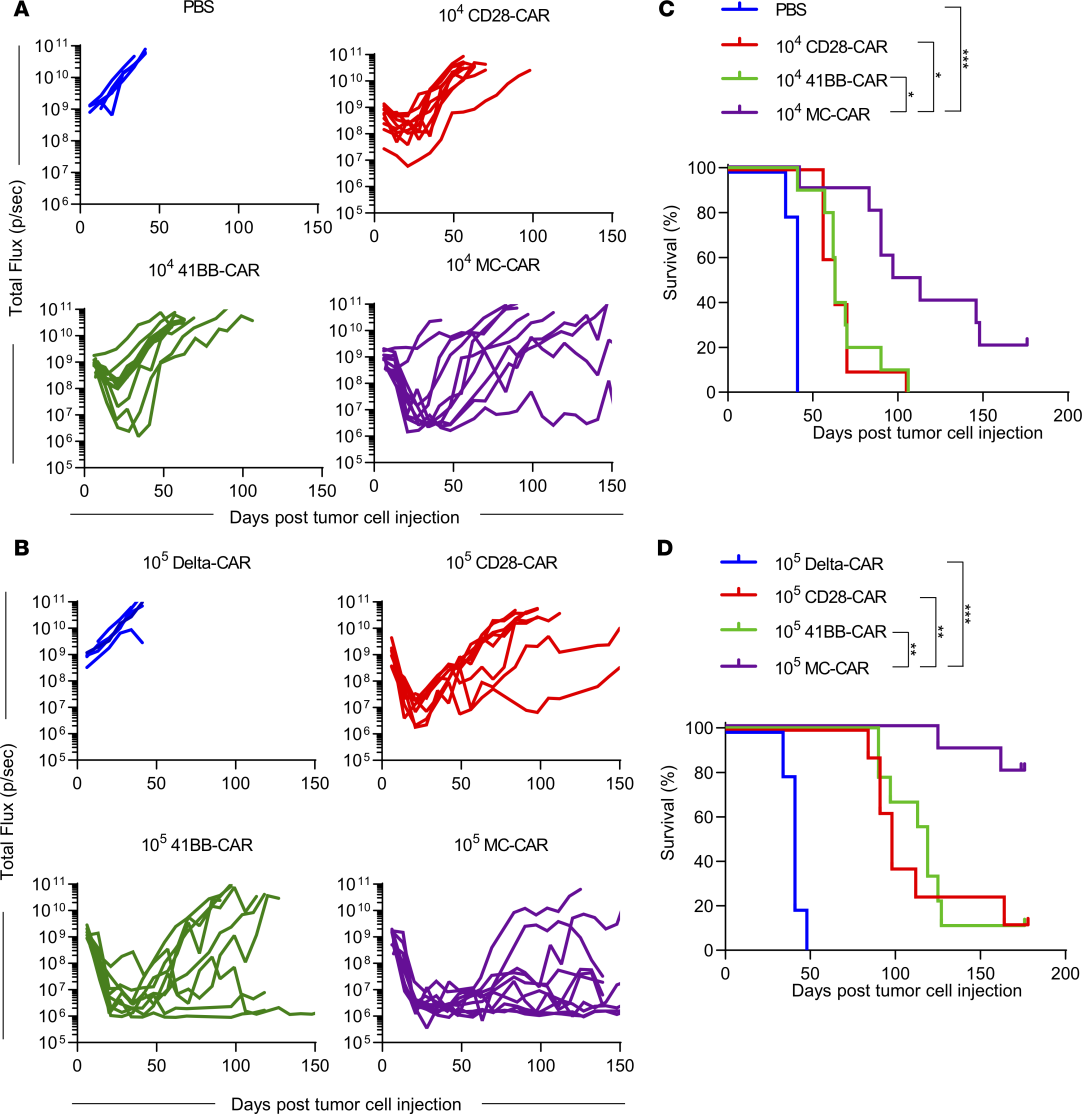
MC-CAR T cells exhibit superior antitumor activity in i.p. LM7 model. (**A**–**D**) NSG mice were injected with 1 × 10^6^ LM7-ffLuc i.p. One week later, mice were injected with 1 × 10^4^ or 1 × 10^5^ CD28-, 41BB-, or MC-CAR T cells. PBS and 1 × 10^5^ Delta-CAR T cells were used as controls. (**A**) Total flux from tumor cells in all mice treated with 1 × 10^4^ EphA2 CAR T cells (PBS: *n* = 5; CD28, 41BB, MC: *n* = 10). (**B**) Total flux from tumor cells in all mice treated with 1 × 10^5^ EphA2 CAR T cells (Delta: *n* = 5, CD28: *n* = 8, 41BB: *n* = 9, MC: *n* = 10). (**C** and **D**) Kaplan-Meier survival analysis of mice treated with 1 × 10^4^ (**C**) or 1 × 10^5^ (**D**) EphA2 CAR T cells (log-rank Mantel-Cox test with Bonferroni’s correction for multiple comparisons; *P < 0.05; **P < 0.01; ***P < 0.001). Experiments were repeated twice with CAR T cells generated from 2 different healthy donors.

We evaluated the antitumor activity of CAR T cells in vivo against the LM7 cell line. NOD/SCID IL-2Rγ–null (NSG) mice were injected i.p. with 1 × 10^6^ LM7 cells genetically modified to express GFP-firefly luciferase (GFP.ffLuc). Seven days later, mice were injected with 1 × 10^5^ or 1 × 10^4^ CAR T cells i.p. Control mice received 1 × 10^5^ Delta-CAR T cells or no T cells (PBS). Tumor burden was monitored by serial bioluminescence imaging (Figure 3, A and B; Supplemental Figure 9, A and B). At each dose level, CD28-, 41BB-, and MC-CAR T cells induced tumor regression, resulting in a significant survival advantage (*P* < 0.001) in comparison with control mice (Figure 3, C and D). In addition, there was a significant survival advantage (1 × 10^4^ T cells: *P* < 0.05, 1 × 10^5^ T cells: *P* < 0.01) for mice that received MC-CAR T cells (median survival: 1 × 10^4^ T cells: 105 days; 1 × 10^5^ T cells: undefined) in comparison with mice that received CD28-CAR (median survival: 1 × 10^4^ T cells: 63 days; 1 × 10^5^ T cells: 98 days) or 41BB-CAR T cells (median survival: 1 × 10^4^ T cells: 63 days; 1 × 10^5^ T cells: 113 days) (Figure 3, C and D). For each CAR T cell group, at least 4 mice with recurrent tumors were evaluated for antigen loss variants. All tumors remained EphA2 positive (Supplemental Figure 10). In order to exclude the possibility that the enhanced antitumor activity of MC-CAR T cells is in part due to tumor cell recognition by the endogenous αβ TCR of MC-CAR T cells, we generated (a) MC-CAR T cells in which the TCR α constant (TRAC) region was knocked out by CRISPR/Cas9 gene editing (MC TRAC KO-CAR T cells) or (b) MC-CAR T cells that expressed an MC-CAR in which the 6 tyrosines in the immunoreceptor tyrosine-based activation motifs (ITAMs) of the ζ-signaling domain were replaced with phenylalanine (MC mutITAM-CAR). TRAC KO was efficient as judged by more than 85% αβ TCR–negative CAR T cells, and TRAC KO did not affect CAR expression (Supplemental Figure 11, A and B). Knocking out TRAC in MC-CAR T cells did not impair their ability to kill U373 or LM7 in a 24-hour MTS assay in vitro or their antitumor activity in the LM7 model in vivo. In contrast, MC mutITAM-CAR T cells had no antitumor activity in vitro and in vivo (Supplemental Figure 11, C–E). Thus, the observed antitumor activity of MC-CAR T cells depends on the expression of a functional CAR and does not rely on endogenous αβ TCR expression.

### MC-CAR T cells have greater proliferative capacity and persistence than CD28- and 41BB-CAR T cells in vivo.

**Figure 4:**
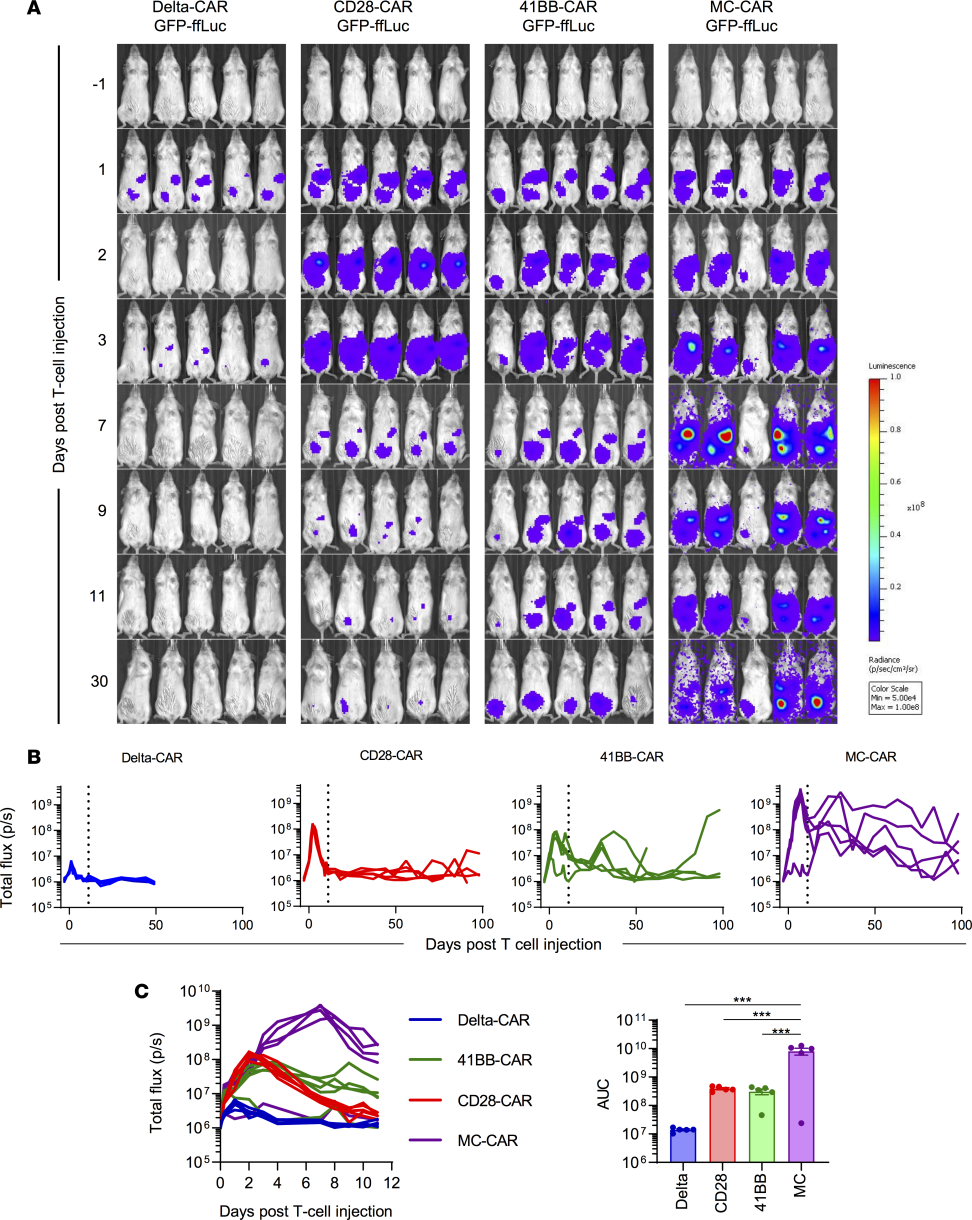
MC-CAR T cells expand more and persist longer in vivo than CD28 or 41BB CAR T cells. NSG mice were injected with 1 × 10^6^ LM7 i.p. One week later they were injected with 1 × 10^5^ T cells that had been doubly transduced to express a CAR and ffLuc. (**A**) Bioluminescence imaging corresponding to T cell expansion/persistence after injection into i.p. LM7-bearing mice (*n* = 5 mice/group). (**B**) Quantitative bioluminescence data from LM7-bearing mice treated with CAR-ffLuc T cells. (**C**) Bioluminescence data until day 11 after T cell injection (denoted by vertical line in **B**), corresponding to peak of T cell expansion, as well as area under the curve (AUC) analysis to evaluate CAR T cell exposure (*n* = 5, mean ± SEM, 1-way ANOVA with Tukey’s test for multiple comparisons, ***P < 0.001).

Given their superior antitumor activity, we hypothesized that MC-CAR T cells would proliferate more and persist longer in vivo. We therefore tracked T cells in the same animal model by bioluminescence imaging, using CAR T cells transduced with GFP-ffLuc (Supplemental Figure 12A) and unmodified LM7 cells (Figure 4, A–C). Median expansion of MC-CAR T cells was 198.4-fold (peak flux within 11 days/baseline flux 6 hours after T cell injection), followed by CD28-CAR (27-fold), 41BB-CAR (8.3-fold), and Delta-CAR (2.70-fold) T cells (Figure 4, A–C). This greater expansion resulted in a significantly greater CAR T cell (*P* < 0.001) exposure of mice infused with MC-CAR T cells as judged by AUC analysis (Figure 4C). Mice were followed out to 100 days after T cell injection, revealing longer persistence of MC-CAR T cells (Figure 4B) without clinical signs (severe weight loss, fur loss) of graft-versus-host disease.

### MC-CAR T cells exhibit baseline activation and retain a less differentiated state after antigen-specific stimulation in comparison with CD28- and 41BB-CAR T cells.

**Figure 5:**
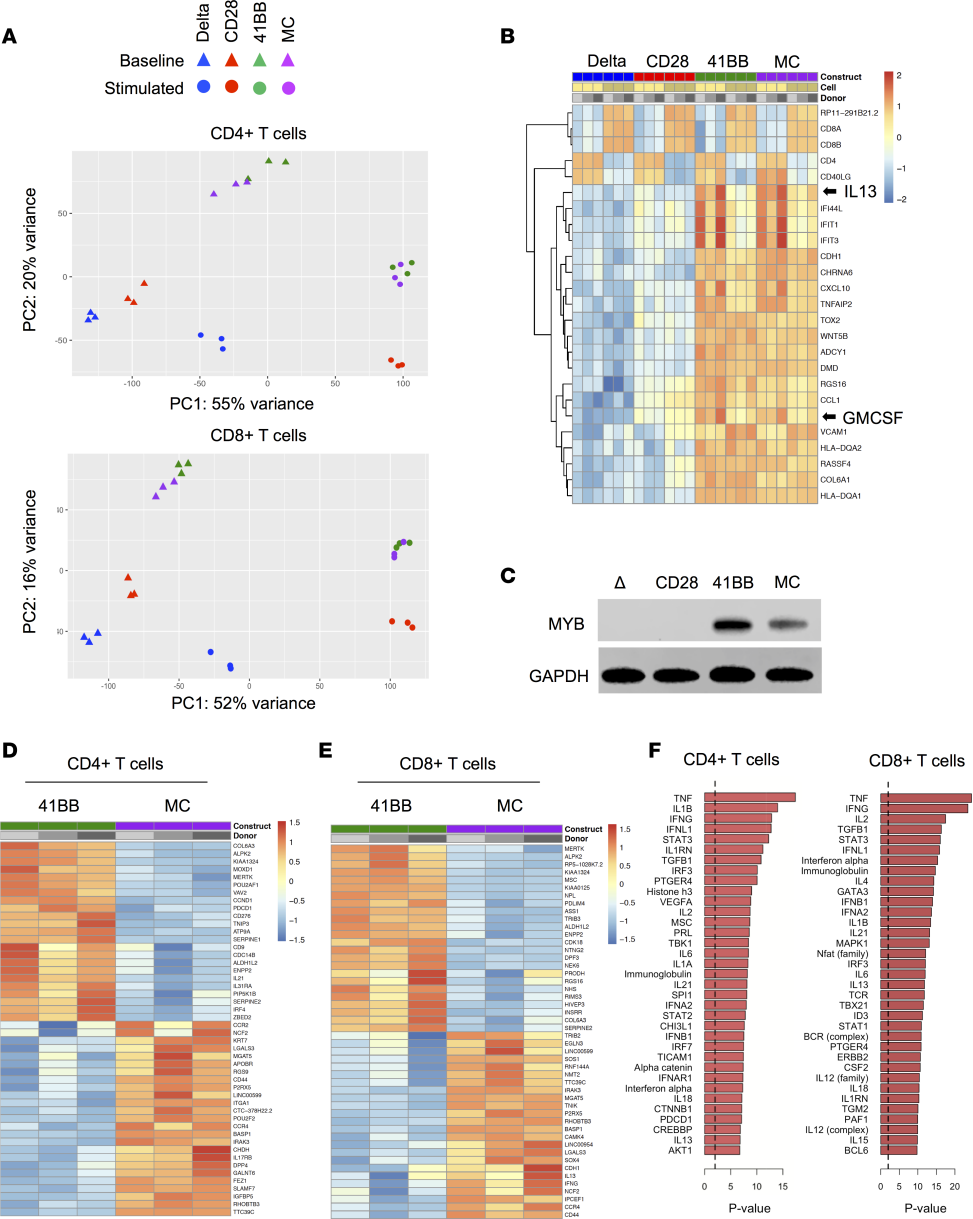
FOXM1 and MYB pathways are upregulated in MC-CAR and 41BB-CAR T cells. CAR T cells from 3 healthy donors were sorted into CD4^+^CAR^+^ and CD8^+^CAR^+^ subsets. RNA was extracted from sorted cells and sequenced on an Illumina NovaSeq platform. (**A**) Principal components analysis of the 3000 most variable genes. (**B**) Top 25 differentially expressed genes at baseline in CD4^+^ and CD8^+^ CAR T cells. (**C**) Western blot showing expression of MYB in unstimulated CAR T cells. GAPDH was used as a loading control. (**D** and **E**) Top 25 differentially expressed genes in each direction for unstimulated CD4^+^ (**D**) and CD8^+^ (**E**) 41BB- vs. MC-CAR T cells. (**F**) Genes differentially expressed between 41BB- and MC-CAR T cells were examined to identify upstream regulatory molecules and mechanisms that differentiate them using Ingenuity Pathway Analysis (IPA). The top 35 upstream regulators, ranked by *P* value, identified using IPA are shown. Enlarged versions of **B** and **D**–**F** are provided in Supplemental Figures 18–21.

We performed RNA-Seq analysis to compare CD28-, 41BB-, and MC-CAR T cells because we had not observed significant phenotypic differences between CAR T cell populations. Seven to 10 days after initial activation and transduction, CD28-, 41BB-, or MC-CAR T cells from 3 donors were either sorted directly or first stimulated with LM7 cells for 24 hours before sorting into CD4^+^ or CD8^+^ CAR T cells. Sorted cells were subjected to library preparation and next-generation sequencing. Unstimulated and stimulated sorted Delta-CAR T cells served as controls. Principal components analysis of the top 3000 most variable genes revealed at baseline 2 CAR T cell groups: (a) Delta- and CD28-CAR T cells and (b) 41BB- and MC-CAR T cells (Figure 5A). After stimulation, 41BB- and MC-CAR T cells continued to group together, whereas CD28-CAR T cells separated from Delta-CAR T cells.

In CD4^+^ and CD8^+^ cells, we identified 7518 and 5048 differentially expressed genes, respectively, at a 5% false discovery rate and a log fold change magnitude greater than 1 that differentiated Delta- and CD28-CAR T cells from 41BB- and MC-CAR T cells. The top differentially expressed genes between the 2 groups revealed genes indicative of baseline 41BB- and MC-CAR T cell activation, including *CSF2* (GM-CSF) and *IL13* expression (Figure 5B). ChIP enrichment analysis (ChEA) revealed that differentially expressed genes were significantly enriched for *MYB* (*P* < 1 × 10^–30^) or *FOXM1* (*P* = 5 × 10^–28^) targets (Table 1). Both MYB and FOXM1 are cell cycle master regulators, and we supported differential MYB expression by Western blot analysis (Figure 5C). Two of the top 3 enriched GO categories were associated with DNA replication (*P* = 3.4 × 10^–13^) and sister chromatid adhesion (*P* = 1.3 × 10^–11^) (Table 1). Thus, 41BB- and MC-CAR T cells expressed higher levels of genes that are important for cell division and proliferation. While 41BB- and MC-CAR T cells formed 1 cluster as judged by principal components analysis, 1202 genes were differentially expressed between CD4^+^ 41BB- and MC-CAR T cells and 620 genes between CD8^+^ 41BB- and MC-CAR T cells, respectively (Figure 5, D and E; Supplemental Tables 1 and 2). These differentially expressed genes were examined to identify upstream regulatory molecules and mechanisms that differentiate 41BB- and MC-CAR T cells using Ingenuity Pathway Analysis (IPA) (Figure 5F) (22). Top differentially expressed genes included proinflammatory cytokines, including TNF-α, IL-1β, and IFN-γ.

**Figure 6:**
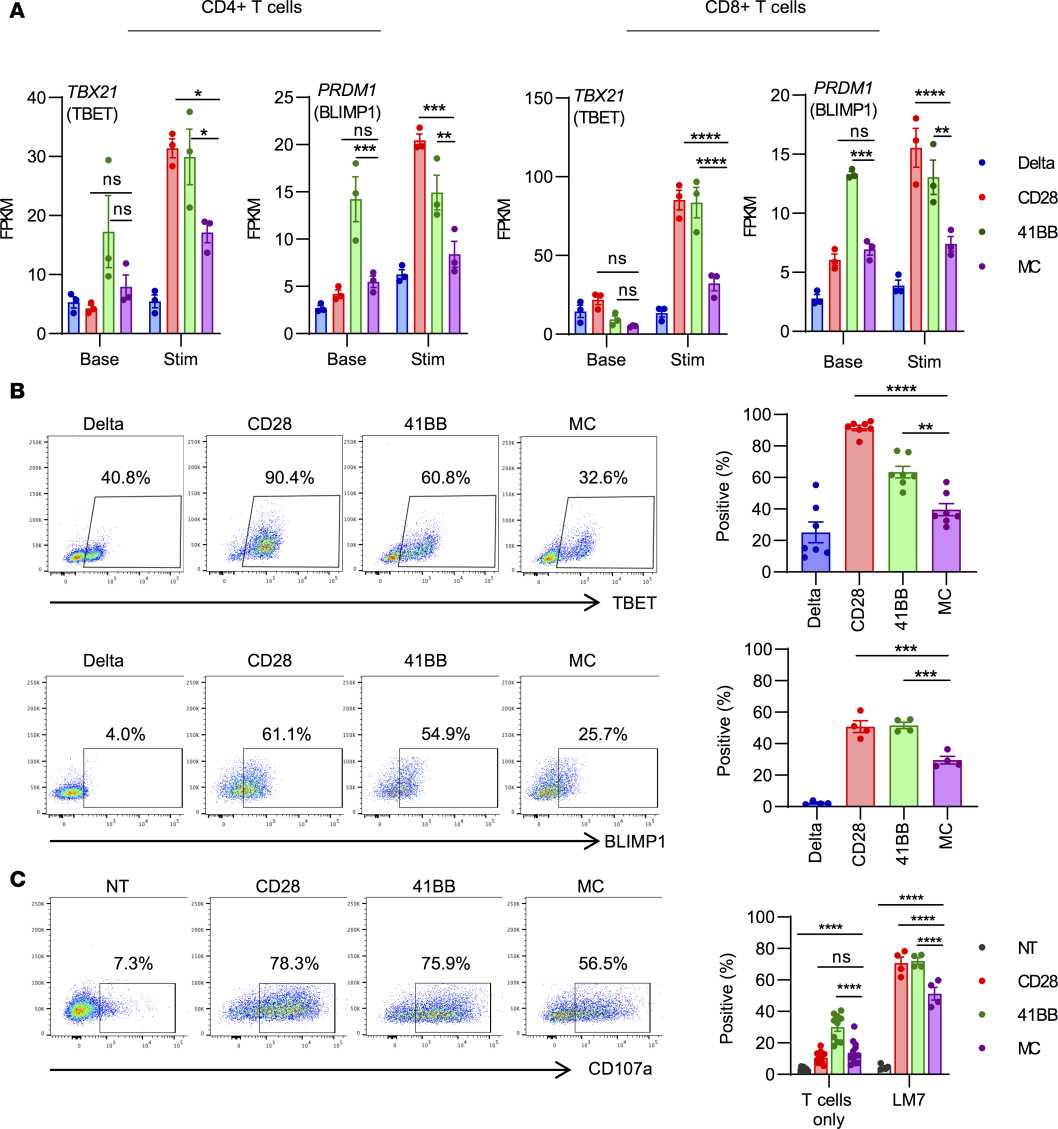
MC-CAR T cells retain a less differentiated phenotype postactivation. (**A**) CAR T cells from 3 healthy donors were taken directly from culture or first stimulated with LM7 tumor cells for 24 hours before being sorted into CD4^+^CAR^+^ and CD8^+^CAR^+^ subsets. RNA was extracted from sorted cells and sequenced on an Illumina NovaSeq platform. Expression of *PRDM1* (encodes BLIMP1) and *TBX21* (encodes TBET) at baseline and after 24-hour stimulation with LM7 in CD4^+^ (left) and CD8^+^ (right) T cells (*n* = 3, mean ± SEM, 2-way ANOVA with Tukey’s test for multiple comparisons). FPKM, fragments per kilobase of transcript per million mapped reads. (**B**) CAR T cells were stimulated with rhEphA2 protein for 24 hours before intracellular staining for the transcription factors TBET and BLIMP1. Representative flow plots and summary of expression for all donors are shown (TBET: *n* = 7, BLIMP1: *n* = 4, mean ± SEM, 1-way ANOVA with Tukey’s test for multiple comparisons). (**C**) T cells were incubated with LM7 tumor cells at a 1:2 E/T ratio for 5 hours in the presence of GolgiStop and stained for CD107a, a surrogate marker for degranulation. Representative flow plots and summary expression for all donors are shown (T cells only: *n* = 13, T cells vs. LM7: *n* = 4, mean ± SEM, 2-way ANOVA with Tukey’s test for multiple comparisons). All statistical tests were performed in comparison with MC-CAR T cells (*P < 0.05; **P < 0.01; ***P < 0.001; ****P < 0.0001). Enlarged versions of **A** and **B** are provided in Supplemental Figures 22 and 23.

**Figure 7:**
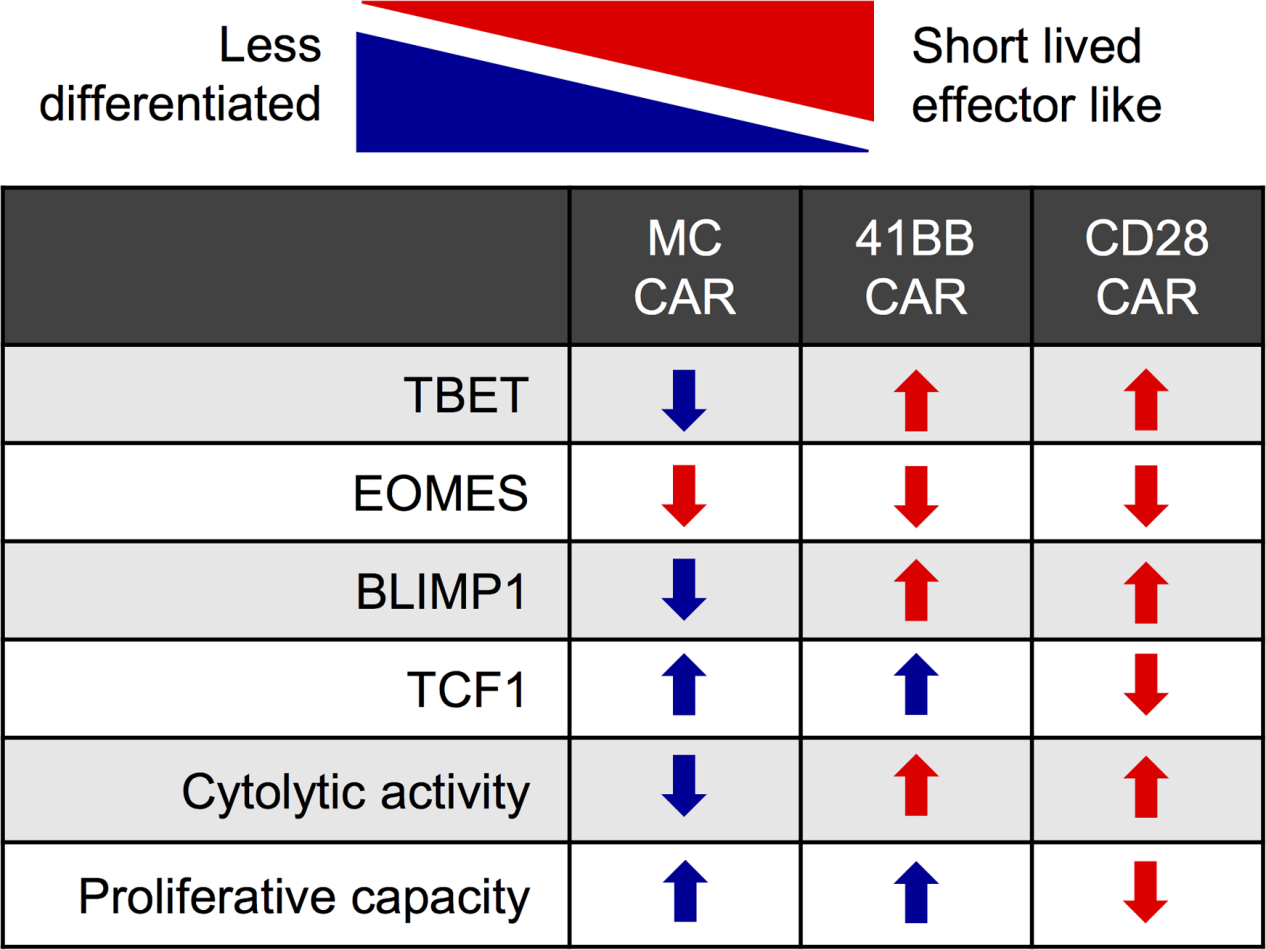
Graphical summary of results. Transcription factors drive T cell differentiation, which is accompanied by phenotypic changes. A graphical description of CD28-, 41BB-, and MC-CAR T cells is shown. Blue indicates phenotype consistent with less differentiation, red with more differentiation. Up arrows indicate higher expression, down arrows lower expression.

After stimulation, MC-CAR T cells expressed distinct sets of genes at higher or lower levels in comparison with CD28- and 41BB-CAR T cells. We identified 5145 (CD4^+^ T cells) and 908 (CD8^+^ T cells) differentially expressed genes comparing MC- and CD28-CAR T cells and 992 (CD4^+^ T cells) and 454 (CD8^+^ T cells) genes comparing MC- and 41BB-CAR T cells (Supplemental Figure 13, A and B; and Supplemental Table 3). Gene Set Enrichment Analysis (GSEA) of MC- and 41BB-CAR T cells, after removing baseline expression differences, revealed enrichment for mammalian target of rapamycin complex 1 (mTORC1) signaling and MYC targets in MC-CAR T cells, 2 pathways that are critical for T cell metabolism (Supplemental Figure 13C). Targeted analysis of transcription factors that play a pivotal role in T cell differentiation, T cell transcription factor 7 (TCF7), TBET, eomesodermin (EOMES), and BLIMP1, revealed that MC-CAR T cells expressed significantly lower levels of *TBX21* (TBET) and *PRDM1* (BLIMP1) than CD28- and 41BB-CAR T cells after stimulation (Figure 6A), which we supported by flow cytometry (Figure 6B). RNA-Seq analysis revealed higher levels of *TCF7* expression in 41BB- and MC-CAR T cells than CD28-CAR T cells after stimulation, although it was not statistically significant. Intracellular flow cytometry supported this finding at the protein level, this time reaching statistical significance (Supplemental Figure 14, A and B). In contrast, no difference in EOMES expression was observed (Supplemental Figure 14, A and B). Higher levels of TBET and BLIMP1 expression in CD28- and 41BB-CAR T cells suggest that these cells differentiate into short-lived effector T cells after stimulation in contrast with MC-CAR T cells. Consistent with this observation, CD28- and 41BB-CAR T cells expressed higher levels of granzyme B (Supplemental Figure 14D) and CD107a, a surrogate marker for degranulation, after antigen-specific simulation (Figure 6C). Thus, MyD88.CD40 costimulation restrains activation-induced differentiation, resulting in a less effector-like phenotype with decreased cytotoxicity but increased proliferative capacity and persistence in comparison with CD28 or 41BB costimulation (Figure 7).

## Discussion

We show here that incorporating MyD88 and CD40 signaling domains into the endodomain of CARs enhances the effector function of CAR T cells both in vitro and in vivo. MC-CAR T cells had superior effector function as judged by their ability to expand and retain their cytolytic activity after repeated exposure to tumor cells in comparison with CD28- or 41BB-CAR T cells. Transcriptomic analysis revealed that these functional differences were accompanied by changes in gene expression. MC-CAR T cells displayed higher levels of transcription factors associated with the maintenance of a stem-like phenotype in T cells and low expression of transcription factors that drive T cell differentiation.

We successfully generated T cells expressing all 3 CARs and observed no significant differences in phenotype or antigen-dependent cytokine production. Cytotoxicity assays revealed that EphA2-CAR T cells with a CD28 endodomain exhibited the most cytolytic activity within 24 hours, which is consistent with previous reports showing it provides a stronger activating signal than 41BB (23). However, CD28-CAR T cells exhibited significant nonspecific killing of U373 EphA2 KO cells in comparison with 41BB- and MC-CAR T cells. Although it is possible that the difference in nonspecific killing resulted from the lower expression of MC-CARs, it is likely that these costimulatory domains require a stronger stimulus to induce killing, which the antigen-negative cells do not provide. Indeed, it has previously been reported that 41BB-CAR T cells exhibit less nonspecific killing than their CD28 counterparts (24), which we also observed for our CD28- and 41BB-CAR T cells despite similar levels of CAR expression. Of note, while CD28-CAR and, to a lesser extent, 41BB-CAR T cells recognized U373 EphA2 KO cells as judged by cytotoxicity assays, U373 EphA2 KO cells did not induce significant cytokine production or expansion of CD28- or 41BB-CAR T cells.

In order to interrogate the performance of CD28-, 41BB-, and MC-CAR T cells upon chronic antigen exposure, we repeatedly exposed them to fresh tumor cells in the absence of exogenous cytokines until they stopped killing. We decided on this assay because it most closely recapitulates the recursive antigen exposure of CAR T cells within solid tumors. MC-CAR T cells proved the most efficacious against 2 EphA2-positive tumor cell lines, expanding more and retaining their cytolytic capacity significantly longer than CD28 or 41BB. We examined the expression of the exhaustion markers PD-1, LAG3, and TIM3 7 days after the first and third stimulations with LM7 tumor cells. Although we saw little difference between CAR T cell populations in LAG3 or TIM3 expression at either time point, PD-1 expression was lower in MC- than CD28-CAR T cells after the first stimulation and lower in comparison with CD28- or 41BB-CAR T cells after the third stimulation.

When we evaluated CD28-, 41BB-, and MC-CAR T cells in vivo, all constructs exhibited potent antitumor activity. However, MC-CAR T cells had greater antitumor activity at both evaluated dose levels, resulting in a significantly improved (*P* < 0.05) overall survival in comparison with CD28- and 41BB-CAR T cells. Antigen loss has widely been reported as a reason for CAR T cell therapy failure, in both clinical and preclinical studies (8, 25, 26). However, all recurrent tumors after CAR T cell therapy, even those that recurred at very late time points (>3 months after T cell injection), still expressed EphA2 at similar intensity in comparison with tumors from control mice. While the absence of antigen escape variants is most likely multifactorial, it is consistent with the central role of EphA2 in maintaining the malignant phenotype of cancer cells (19, 27).

Evaluation of the fate of CD28-, 41BB-, and MC-CAR T cells in vivo offered a potential explanation of the superior antitumor activity of MC-CAR T cells. Consistent with what has been observed in clinical studies with CD19-CAR T cells (2, 3, 25), our CD28-CAR T cells expanded rapidly after injection but quickly contracted, while 41BB-CAR T cells expanded slower but persisted longer. However, MC-CAR T cells expanded the most of all 3 constructs during the first 11 days after T cell injection and could be detected within mice for a significantly longer period. Thus, we observed a correlation between superior persistence and antitumor activity in our model, which has been reported by others in preclinical studies (8, 28, 29). In addition, the durability of responses for CD19^+^ malignancies has also been correlated to CAR T cell persistence in humans (25). Since signaling through the endogenous αβ TCR has the potential to confound the in vitro and in vivo analysis of CAR T cells (30), we generated MC-CAR T cells in which the endogenous αβ TCR was knocked out (TRAC KO) or that expressed a nonfunctional MC-CAR (MC mutITAM-CAR). MC TRAC KO-CAR T cells had similar antitumor activity to unmodified MC-CAR T cells while MC mutITAM-CAR T cells had no antitumor activity, providing strong evidence that the superior activity of MC-CAR T cells is dependent on a functional CAR and not on activation of endogenous αβ TCRs.

To evaluate if the benefit of MC costimulation can be extended to other CARs, we designed an MC-CAR with an HER2-specific scFv. T cells expressing this CAR had decreased effector function (Supplemental Figure 15), which is similar to findings by other investigators, who evaluated a CD19-specific MC-CAR (14). To overcome this obstacle, we generated T cells expressing an HER2-CAR and an EphA2-CAR that only contained an MC costimulatory endodomain. Provision of MC costimulation in a separate molecule did not interfere with ζ signaling and endowed CAR T cells with superior effector function (Supplemental Figure 16). Studies are in progress to further delineate the structure/function relationship of MC-CARs. In addition, we have recently designed chimeric cytokine receptors that directly link MyD88 signaling to CAR T cell activation to overcome the structural limitation of directly incorporating MyD88 signaling domains into CARs (31).

We performed RNA-Seq to decipher differences in gene expression between Delta-, CD28-, 41BB-, and MC-CAR constructs. Principal components analysis as well as differential expression of activation-associated genes demonstrated that 41BB- and MC-CAR T cells exhibited baseline activation (aka tonic signaling), which has been previously reported for 41BB-CARs (32). Contrary to other reports (33), we observed minimal evidence of baseline signaling in our CD28-CAR T cells, highlighting that other structural components of CARs contribute to their signaling characteristics. Tonic signaling in CD28- and 41BB-CAR T cells has previously been shown to be associated with T cell exhaustion and inferior antitumor activity (32, 33). Given that baseline signaling in MC-CAR T cells is paired with superior antitumor activity, our results suggest that tonic signaling by itself might not be detrimental to T cell function. Pathway analysis of unstimulated CAR T cells revealed high expression of MYB-associated genes in 41BB- and MC-CAR T cells. It has recently been reported that c-Myb maintains a stem-like phenotype in CD8^+^ murine T cells, by promoting T cell survival and by restraining T cell differentiation (34). These phenotypes are driven at least partially by promoting the expression of the antiapoptotic molecule Bcl-2, and Tcf-7, a transcription factor that enhances and maintains memory formation and self-renewal, while restraining Zeb2, a transcription factor that promotes terminal CD8^+^ T cell differentiation (34–38). Consistent with this, we observed higher expression of the antiapoptotic proteins Bcl-2 and Bcl-xL in MC-CAR T cells after stimulation. While we observed no differences in the RNA expression of *ZEB2* (GSE158144), CD4^+^ MC-CAR T cells expressed higher levels of *TCF7* at baseline in comparison with 41BB-CAR T cells. After antigen-specific activation, 41BB- and MC-CAR T cells expressed higher levels of TCF7 in comparison with CD28-CAR T cells. This suggests that 41BB- and MC-CAR T cells have enhanced potential for self-renewal in comparison with CD28-CAR T cells, which was mirrored by improved persistence in vivo.

In our gene expression analysis, we identified 2 other differentially expressed transcription factors, *BLIMP1* and *TBET*, that promote terminal differentiation of CD8^+^ T cells (39). For example, Blimp-1 promotes terminal effector differentiation and represses memory formation in murine models of chronic viral infection (40–42). Similarly, high levels of T-bet promote the development of short-lived effector CD8^+^ murine T cells, whereas low levels promote memory precursor development (43, 44). Antigen-specific stimulation induced high levels of expression of both TBET and BLIMP1 in CD28- and 41BB-, but not MC-CAR, T cells. MC-CAR T cells also exhibited a lower cytolytic potential, corroborating a less differentiated state, which correlates with improved efficacy of adoptively transferred T cells in other models (45–47).

The majority of our studies were performed with a CAR containing both MyD88 and CD40, making it difficult to evaluate the individual contributions of MyD88 and CD40. We therefore generated CARs with a MyD88.ζ (MyD88-CAR) or CD40.ζ (CD40-CAR) endodomain and tested them in our repeat stimulation assay (Supplemental Figure 17). CD40-CARs endowed T cells with a significantly lower ability to expand and sequentially kill target cells. In contrast, there was no significant difference in expansion between MyD88-CAR and MC-CAR T cells; however, MC-CAR T cells had a substantially greater ability to sequentially kill target cells. Thus, CD40 signaling by itself is not sufficient but in the context of MyD88 activation enhances the effector function of CAR T cells. The critical role of MyD88 signaling is also supported by our RNA-Seq data set because one of the top 3 differentially expressed GO categories in MC-CAR T cells was response to viruses. While our study demonstrates that MyD88 signaling improves CAR T cell function, it also has limitations that have to be addressed in future studies. For example, we did not perform mechanistic studies to demonstrate causality of identified pathways like knocking our transcription factors (e.g., MYB) or molecules that are part of mTORC1.

In conclusion, MC costimulation results in a less differentiated CAR T cell phenotype (Figure 7), increasing proliferative capacity and persistence. These findings support early-phase clinical testing of our approach, especially because T cells expressing conventional CARs with CD28 or 41BB costimulatory endodomains have had limited activity against solid tumors in the clinic.

## Methods

### Tumor cell lines.

The glioma cell line U373 and B cell leukemia cell line BV173 were purchased from the American Type Tissue Collection (ATCC) and the German Collection of Microorganisms and Cell Cultures, respectively. The metastatic osteosarcoma cell line LM7 was provided by Eugenie Kleinerman (MD Anderson Cancer Center, Houston, Texas, USA) in 2011. The generation of LM7.eGFP.ffLuc was previously described (12). U373 EphA2 KO cells were generated using CRISPR/Cas9. A guide RNA targeting the sequence GGGGGGCCGCTCACCCGCAA was selected using the online CRISPRscan scoring algorithm (48) to maximize cutting efficiency and minimize off-target effects. sgRNA was generated using the HiScribe T7 *In Vitro* Transcription Kit (New England Biolabs). U373 cells were electroporated with 1 μg Cas9 with NLS (PNA Bio) and 1 μg sgRNA using a Neon Transfection System (Thermo Fisher Scientific). All cell lines were grown in DMEM or RPMI (Cytiva) supplemented with 10% fetal bovine serum (FBS; Cytiva) and 2 mM Glutamax (Invitrogen, Thermo Fisher Scientific). Cell lines were authenticated using the ATCC’s human STR profiling cell authentication service.

### Generation of retroviral vectors.

The generation of the SFG retroviral vectors encoding EphA2-CARs with a CD28 or 41BB costimulatory domain, a nonfunctional EphA2-CAR without a signaling domain (Delta-CAR), and GFP.ffluc has been previously described (21). In-Fusion cloning (Takara Bio) was used to generate the EphA2-CAR with a MyD88.CD40 costimulatory endodomain without the Toll/interleukin-1 (IL-1) receptor domain using a previously published retroviral vector encoding MyD88.CD40 as a template (12). In-Fusion cloning was used to generate the tCD19.IRES.EphA2-CARs, using a previously published vector encoding FMC63-CD3.IRES.mOrange as the template for the IRES (49). EphA2 MyD88 (MyD88-CAR) and EphA2 CD40 (CD40-CAR) CARs were generated by In-Fusion cloning from the original EphA2 MyD88.CD40 CAR vector. Retroviral particles were generated by transient transfection of HEK293T cells (ATCC) with the EphA2-CAR encoding SFG retroviral vectors, Peg-Pam-e plasmid encoding MoMLV gag-pol, and a plasmid encoding the RD114 envelope protein. Supernatants were collected after 48 hours, filtered, and snap-frozen for later transduction of T cells.

### Generation of CAR T cells.

Human peripheral blood mononuclear cells (PBMCs) were obtained from whole blood of healthy donors under IRB-approved protocols at St. Jude Children’s Research Hospital (SJCRH). To generate CAR T cells, we isolated PBMCs by Lymphoprep (Abbott Laboratories) gradient centrifugation and then stimulated on non–tissue culture–treated 24-well plates, which were precoated with CD3 and CD28 antibodies (αCD3/αCD28; CD3: OKT3, CD28: 15E8; Miltenyi Biotec). rhIL-7 and rhIL-15 (IL-7: 10 ng/mL; IL-15: 5 ng/mL; PeproTech) were added to cultures the next day. On day 2, CD3/CD28-stimulated T cells (2.5 × 10^5^ cells/well) were transduced with RD114-pseudotyped retroviral particles on RetroNectin-coated (Clontech) plates in the presence of IL-7 and IL-15. On day 5, transduced T cells were transferred into new tissue culture 24-well plates and subsequently expanded with IL-7 and IL-15. NT T cells were prepared in the same way minus the addition of retrovirus. CAR T cell expression was determined using flow cytometry at multiple time points posttransduction to ensure continued CAR expression. All experiments were performed 7–21 days posttransduction. Biological replicates were performed using PBMCs from different healthy donors.

### TRAC KO-CAR T cells.

PBMCs were activated with αCD3/αCD28 on day 0, IL-7 and IL-15 were added to cultures on day 1, and T cells were electroporated with ribonucleoproteins (RNPs) targeting TRAC on day 2 using the Neon Transfection System (Thermo Fisher Scientific) and transduced with CAR-encoding retroviral vectors on day 3. sgRNA was designed to target the previously published sequence CAGGGTTCTGGATATCTGT (50). RNPs were precomplexed at an sgRNA/Cas9 ratio of 4.5:1, prepared by adding 3 μL 60 μM sgRNA (Synthego) to 1 μL 40 μM Cas9 (Macro Lab, University of California, Berkeley) and frozen for later use. A total of 6 × 10^5^ T cells were resuspended in 20 μL R buffer and added to 4 μL RNP. Next, 10 μL cells and RNP were electroporated with 3 pulses of 10 ms at 1600 V. Two 10 μL electroporation reactions were pooled in 1 well of a 48-well tissue culture–treated plate containing RPMI + 20% FBS with IL-7 and IL-15. Three to 4 days after transduction, the FBS concentration was reduced to 10% in the T cell culture media.

### Flow cytometry.

A FACSCanto II (BD) instrument was used to acquire flow cytometry data, which were analyzed using FlowJo v10. For surface staining, samples were washed with and stained in PBS (Lonza) with 1% FBS (Cytiva). Intracellular staining was performed using the Foxp3/Transcription Factor Staining Buffer Set (Invitrogen, Thermo Fisher Scientific). For all experiments, matched isotypes or known negatives (e.g., NT T cells) served as gating controls. LIVE/DEAD Fixable Aqua Dead Cell Stain Kit (Invitrogen, Thermo Fisher Scientific) was used as a viability dye.

T cells were evaluated for CAR expression at multiple time points after transduction with CD19-PE (clone J3-119, Beckman Coulter) and anti–human IgG, F(ab′)_2_ fragment specific–Alexa Fluor (AF) 647 (Jackson ImmunoResearch 109-606-006). T cells were phenotyped with several panels at 7–10 days after transduction, 1–7 days after coculture with tumor cells, or 24 hours after stimulation with rhEphA2 using combinations of the following antibodies: TCRαβ-APC (clone T10B9, BD), CD3-APC (clone UTCH1, Beckman Coulter), CD4–Pacific Blue (clone SK3, BioLegend) CD8–PerCP-Cy5.5 (clone SK1, BioLegend), CD19-PE (clone J3-119, Beckman Coulter), CD19-APC (clone J3-119, Beckman Coulter), CD19-BV421 (clone HIB19, BD), CCR7-AF488 (clone G043H7, BioLegend), CD45RA–APC-H7 (clone HI100, BD), LAG3-FITC (clone 11C3C65, BioLegend), TIM3–PE-Cy7 (clone F38-2E2, BioLegend), PD1-PE (clone EH12.2H7, BioLegend), T-bet–AF488 (clone D6N8B, Cell Signaling Technology), TCF1-PE (clone C63D9, Cell Signaling Technology), EOMES–PE-Cy7 (clone WD1928, Invitrogen, Thermo Fisher Scientific), BLIMP1-AF647 (clone 646702, R&D Systems, Bio-Techne), Ki-67–APC (clone Ki-67, BioLegend), Bcl-xL (clone 7B2.5, Invitrogen, Thermo Fisher Scientific), CD69–PE-Cy7 (clone FN50, BioLegend), CD25–APC-H7 (clone M-A251, BD), and 41BB-BV421 (clone 4B4-1, BD).

Tumor cell lines were evaluated for expression of EphA2 with EphA2-APC (clone 371805, R&D Systems, Bio-Techne). Recurrent tumors were removed from mice after they reached our bioluminescence endpoint, homogenized using a gentleMACS Dissociator (Miltenyi Biotec), filtered, and stained with LDA and EphA2-APC.

For the viability assay, T cells were stimulated with recombinant human EphA2 protein for 24 hours or LM7 tumor cells for 7 days, at which point cells were collected, washed with PBS, and stained with LDA and CD19-APC for 30 minutes. Cells were then washed with PBS, resuspended in 1× Annexin V Binding Buffer (BD), and stained with annexin V–PE (BD 556421). After a 15-minute incubation, cells were analyzed on the flow cytometer. LDA and annexin V expression was evaluated on CD19^+^ cells.

For the degranulation assay, tumor cells were labeled with CellTrace Violet (CTV; Invitrogen, Thermo Fisher Scientific) and then incubated with T cells at a 1:2 E/T ratio for 5 hours in the presence of GolgiStop (BD), anti-CD107a-APC (clone H4A3, BD), and anti-CD19–PE (clone J3-119, Beckman Coulter). Cells were then washed and analyzed on the flow cytometer. CD107a expression was evaluated on CTV-CD19^+^ cells.

### Analysis of cytokine production.

A total of 1 × 10^6^ T cells were cocultured with no tumor cells or 5 × 10^5^ LM7, U373, U373 EphA2 KO, or BV173 cells without the provision of exogenous cytokines. After 24 hours, supernatant was collected and frozen for later analysis. Cytokines were measured using a MILLIPLEX MAP Human Cytokine/Chemokine Magnetic Bead Panel (MilliporeSigma) kit on a FLEXMAP 3D System (Luminex).

### MTS assay.

A CellTiter96 AQueous One Solution Cell Proliferation Assay (Promega) was used to assess CAR T cell cytotoxicity. In a tissue culture–treated 96-well plate, 12,500 U373 or 15,000 LM7 cells were cocultured with serial dilutions of T cells to give E/T ratios of 2.5:1, 1.25:1, 0.6:1, and 0.3:1. Media or tumor cells alone served as controls for background and no T cell–mediated cytotoxicity, respectively. Each condition was plated in triplicate. After 24 hours, the media and T cells were removed by gently pipetting up and down to avoid disrupting adherent U373 or LM7 cells. CellTiter96 AQueous One Solution Reagent (MTS + phenazine ethosulfate) in RPMI–10% FBS was added to each well and incubated at 37°C, 5% CO_2_, for 2 hours. The absorbance at 492 nm was measured using an Infinite 200 Pro MPlex plate reader (Tecan) to assess the number of viable cells in each well. Percentages of live tumor cells were determined by the following formula: (sample – media alone)/(tumor alone – media alone) × 100.

### Repeat stimulation assay.

A total of 1 × 10^6^ T cells were cocultured with 5 × 10^5^ tumor cells in a 24-well plate without the provision of exogenous cytokines. Seven days later, the number of T cells was determined with a hemocytometer. If T cells were killed and expanded, 1 × 10^6^ T cells were replated with 5 × 10^5^ fresh tumor cells. This process was repeated weekly until the T cells no longer killed the tumor cells.

### Xenograft mouse models.

Animal experiments followed a protocol approved by the St. Jude Children’s Research Hospital Institutional Animal Care and Use Committee. All experiments used 8- to 12-week female NSG mice purchased from The Jackson Laboratory or obtained from the SJCRH NSG colony. Mice were euthanized when they reached our bioluminescence endpoint of 2 total flux measurements more than 2 × 10^10^ or when they met physical euthanasia criteria (significant weight loss, signs of distress) or when recommended by SJCRH veterinary staff. Mice were injected i.p. with 1 × 10^6^ LM7 cells. Seven days later, 1 × 10^5^ or 1 × 10^4^ CAR T cells were injected i.p. Separate experiments were performed to measure tumor burden or T cell persistence/expansion by using eGFP.ffLuc-expressing tumor cells or T cells, respectively.

### Bioluminescence imaging.

Mice were injected i.p. with 150 mg/kg of d-luciferin 5–10 minutes before imaging, anesthetized with isoflurane (1.5%–2% delivered in 100% O_2_ at 1 L/min), and imaged with a Xenogen IVIS-200 imaging system. The photons emitted from the luciferase-expressing tumor cells were quantified using Living Image software (Caliper Life Sciences). Mice were imaged once per week to track tumor burden and 1–5 times per week to track T cells.

### Western blot.

Cells were washed with PBS and lysed in 1× RIPA Lysis and Extraction Buffer (Thermo Fisher Scientific) + Halt Protease and Phosphatase Inhibitor Cocktail (Thermo Fisher Scientific). Protein quantification was performed using the Pierce BCA Protein Assay Kit (Thermo Fisher Scientific). Samples were prepared by adding Laemmli buffer + β-mercaptoethanol (both from BioRad) to equal volumes of protein and boiling. After SDS-PAGE and wet transfer, the membrane was blocked with 5% milk in TBS-Tween (TBST), then incubated with primary antibodies: goat anti-human EphA2 (R&D Systems, Bio-Techne, AF3035), rabbit anti-human Bcl-2 (clone D55G8, Cell Signaling Technology), rabbit anti-human Myb (clone D2R4Y, Cell Signaling Technology), or mouse anti-human GAPDH (clone 0411, Santa Cruz Biotechnology). The membrane was washed with TBST, then incubated with an appropriate secondary: mouse anti–goat IgG–HRP (sc-2354, Santa Cruz Biotechnology), goat anti–rabbit IgG–HRP (111-036-045, Jackson ImmunoResearch), or goat anti–mouse IgG–HRP (sc-2005, Santa Cruz Biotechnology). The membrane was then washed again before addition of Clarity Western ECL Substrate (Bio-Rad). Membranes were imaged on the Odyssey Fc Imaging System (LI-COR Biosciences). Blots were quantified using Image Studio (LI‑COR Biosciences).

### RNA-Seq.

Delta-, CD28-, 41BB-, and MC-CAR T cells were prepared using PBMCs from 3 healthy donors. At days 7–10 after transduction, T cells were either taken directly from culture (baseline) or cocultured with LM7 at a 2:1 E/T ratio for 24 hours before being prepared for FACS. Cells were sorted on a BD FACSAria III into CD4^+^CD19^+^ or CD8^+^CD19^+^ populations. RNA extraction was performed using a RNeasy Mini Kit (QIAGEN). SJCRH’s Hartwell Center performed library prep and sequencing. mRNA enrichment and cDNA library preparation were performed using the TruSeq Stranded mRNA Library Prep Kit (Illumina). An Agilent TapeStation was used to assess library size and quality. Library concentrations were measured using a Quant-iT PicoGreen dsDNA Assay Kit (Invitrogen, Thermo Fisher Scientific) followed by a low-pass sequencing run on a MiSeq-nano flow cell (Illumina) to estimate cluster generation. The libraries were sequenced on a NovaSeq 6000 using 2 × 100 paired-end sequencing configuration in order to generate approximately 100 million paired reads per sample. RNA-Seq data have been deposited in NCBI’s Gene Expression Omnibus (GEO) and are accessible through GEO series accession number GSE158144.

### Statistics.

For all experiments, the number of biological replicates and statistical analysis test used are described in the figure legend. For comparison between 2 groups, a 2-tailed *t* test was used. For comparisons of 3 or more groups, values were log transformed as needed and analyzed by 1- or 2-way ANOVA with Dunnett’s or Tukey’s posttest. Survival was assessed by the log-rank test with Bonferroni’s adjustment for multiple comparisons. Bioluminescence imaging data were analyzed using 1-way ANOVA or AUC. *P* values less than 0.05 were considered significant. RNA-Seq quality was assessed using fastqc (51). Reads were aligned to hg19 via a pipeline that uses STAR (52) and BWA (53). Aligned reads were counted using HTSeq (54). Genes without at least 10 read counts across unstimulated or stimulated samples were excluded from the analysis. Raw read counts were voom normalized and contrasted using the limma R package using default parameters (55). Log_2_ counts per million values were used for heatmap visualizations and in the GSEAs (56). Heatmaps are *Z*-score scaled by gene.

### Study approval.

Human PBMCs were obtained from whole blood of healthy donors under an IRB-approved protocol at St. Jude Children’s Research Hospital. Animal experiments followed a protocol approved by the St. Jude Children’s Research Hospital Institutional Animal Care and Use Committee.

## Author contributions

BP, GK, and SG conceived and designed the study. BP, PS, and MB acquired data. BP, PS, MB, YF, GK, and SG analyzed and interpreted data. BP, PS, MB, YF, GK, and SG wrote and revised the manuscript. BP, GK, and SG provided administrative, technical, or material support. GK and SG supervised the study.

## Supplementary Material

supplemental data

supplemental Table 1

supplemental Table 2

supplemental Table 3

## Figures and Tables

**Table 1:**
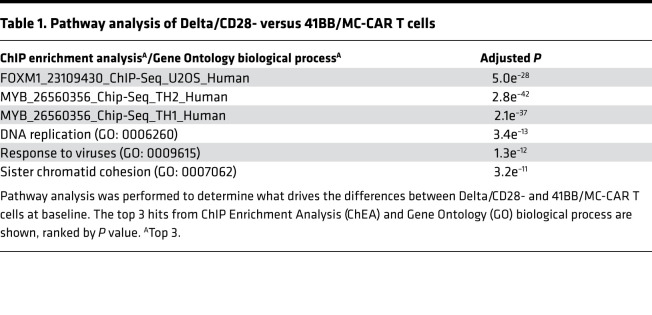
Pathway analysis of Delta/CD28- versus 41BB/MC-CAR T cells

## Version 1 11/05/2020

Electronic publication
