# Supplementary material for: MyD88/CD40 signaling retains CAR T cells in a less differentiated state
Source: JCI Insight. 2020 Nov 5;5(21):e136093. doi: 10.1172/jci.insight.136093 (PMC7710311; doi:10.1172/jci.insight.136093)
Supplement: supplemental data [file jciinsight-5-136093-s042.pdf]

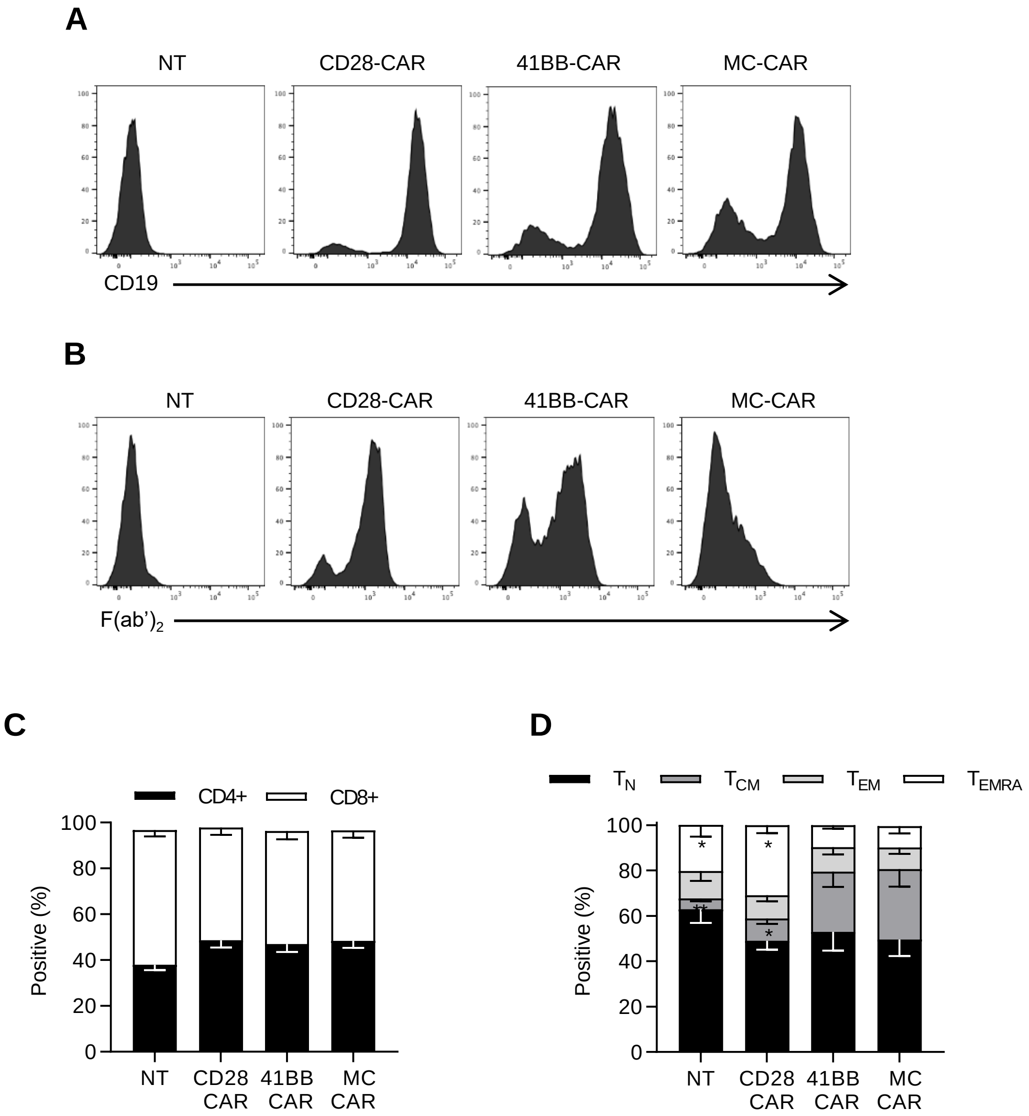

Supplemental Figure 1: Characterization of EphA2-CAR T cells. Representative flow cytometry plots of non-transduced (NT), CD28-CAR, 41BB-CAR, and MC-CAR T cells. CAR T cells were stained with **A.** anti-CD19 or **B.** anti-human IgG, F(ab')$_2$ fragment to detect transduced T cells. **C.** Ratio of CD4 and CD8-positive T cells determined by flow cytometry (n=7, mean + SEM). **D.** CCR7 and CD45RA expression was used to phenotype T cells as naïve-like (T_N: CCR7$^+$CD45RA$^+$), central memory (T_{CM}, CCR7$^+$CD45RA$^-$), effecter memory (T_{EM}, CCR7$^-$CD45RA$^-$), or terminal effector (T_{EMRA}, CCR7$^-$CD45RA$^+$) (n=8, mean + SEM).

Supplemental Figure 1

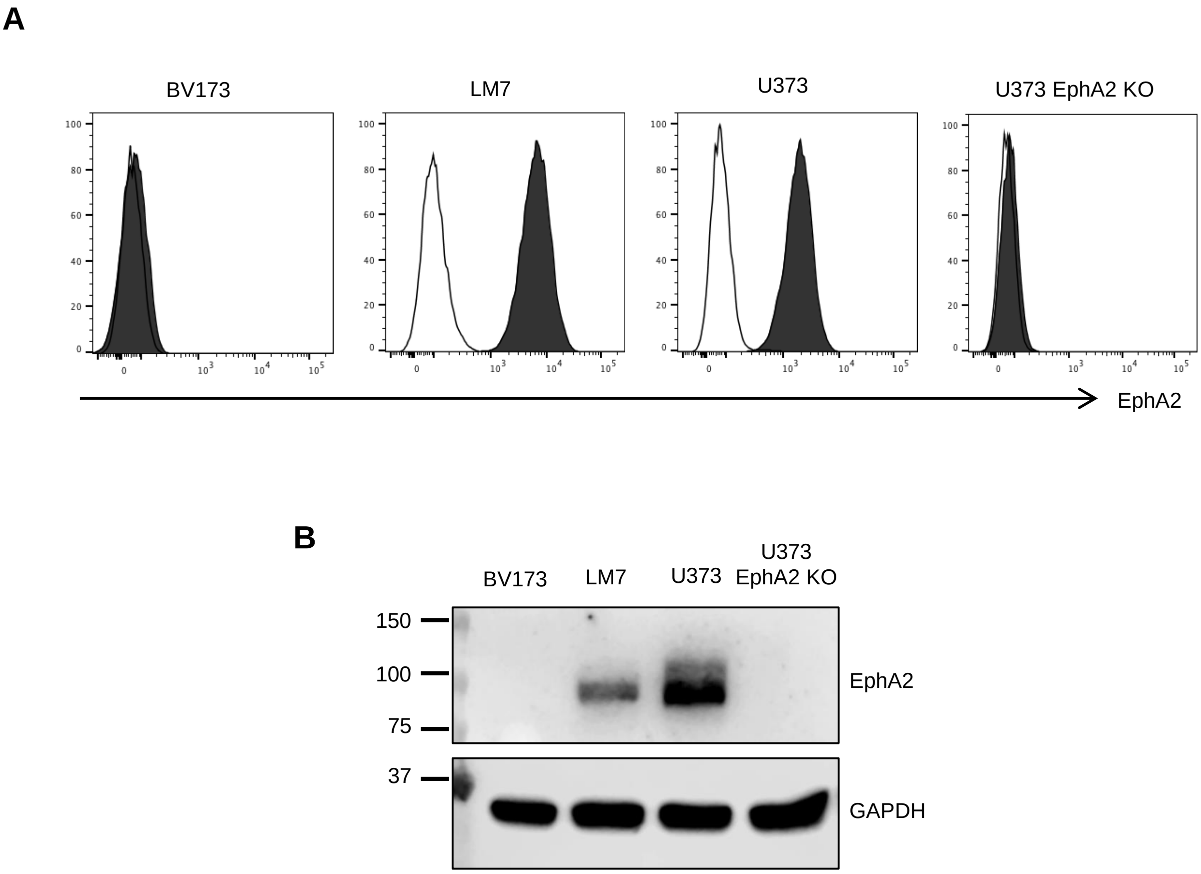

Supplemental Figure 2: EphA2 expression on tumor cell lines used for *in vitro* and *in vivo* assays. **A.** Flow cytometry plots showing expression of EphA2 on tumor cell lines (black filled) with isotype control (not filled). **B.** Western blot was used to confirm expression of EphA2 in whole cell lysates of tumor cells. GAPDH was used as a loading control.

Supplemental Figure 2

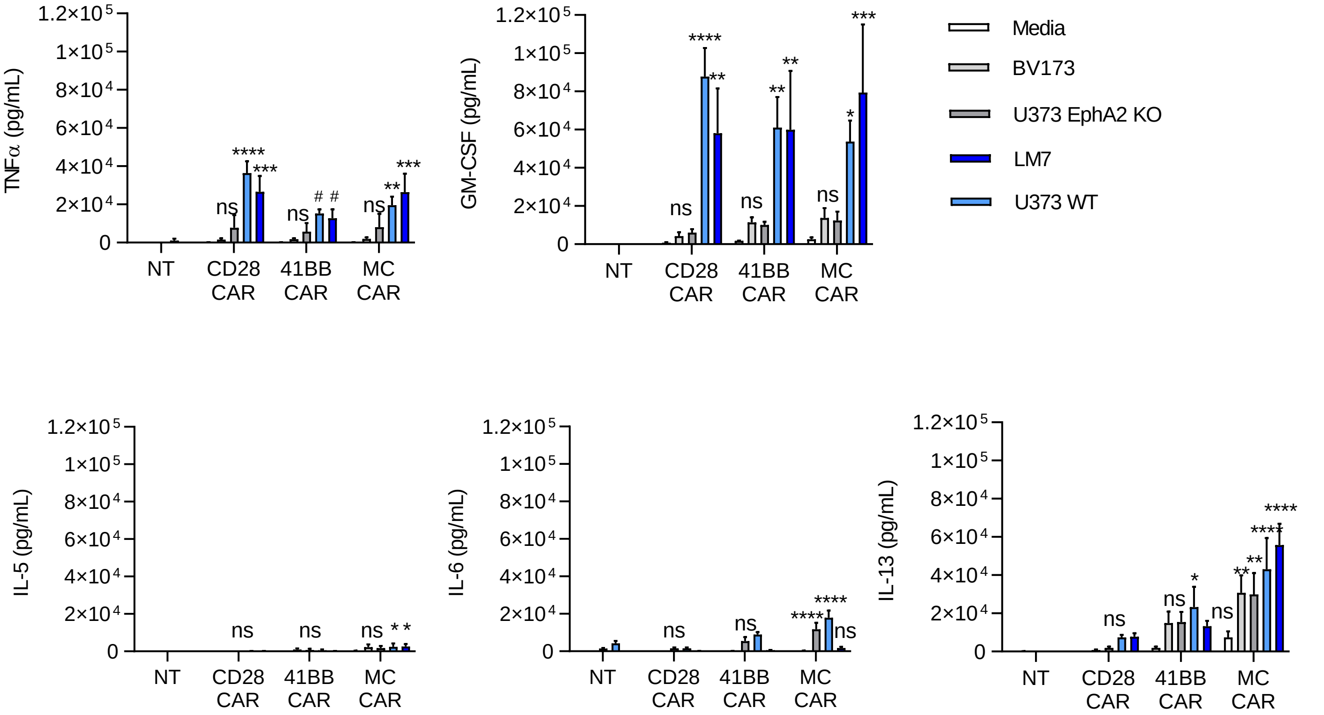

Supplemental Figure 3: Extended cytokine production by CAR T cells. CAR T cells were co-cultured at a 2:1 E:T ratio against EphA2+ (LM7, U373 WT) and EphA2- (BV173, U373 EphA2 KO) cell lines or in media alone (n=5). Supernatant was collected after 24 hours and the production of Th1 (GM-CSF and TNFα) and Th2 (IL-5, IL-6, and IL-13) cytokines was determined using a milliplex assay (n=5, mean + SEM, two-way ANOVA with Dunnett's test for multiple comparisons, all statistical tests are in comparison to NT T cells, ns=not significant; $p<0.1$: #; $p<0.05$: *; $p<0.01$: **; $p<0.001$: ***; $p<0.0001$: ****). Other cytokines are shown in Figure 1.

Supplemental Figure 3

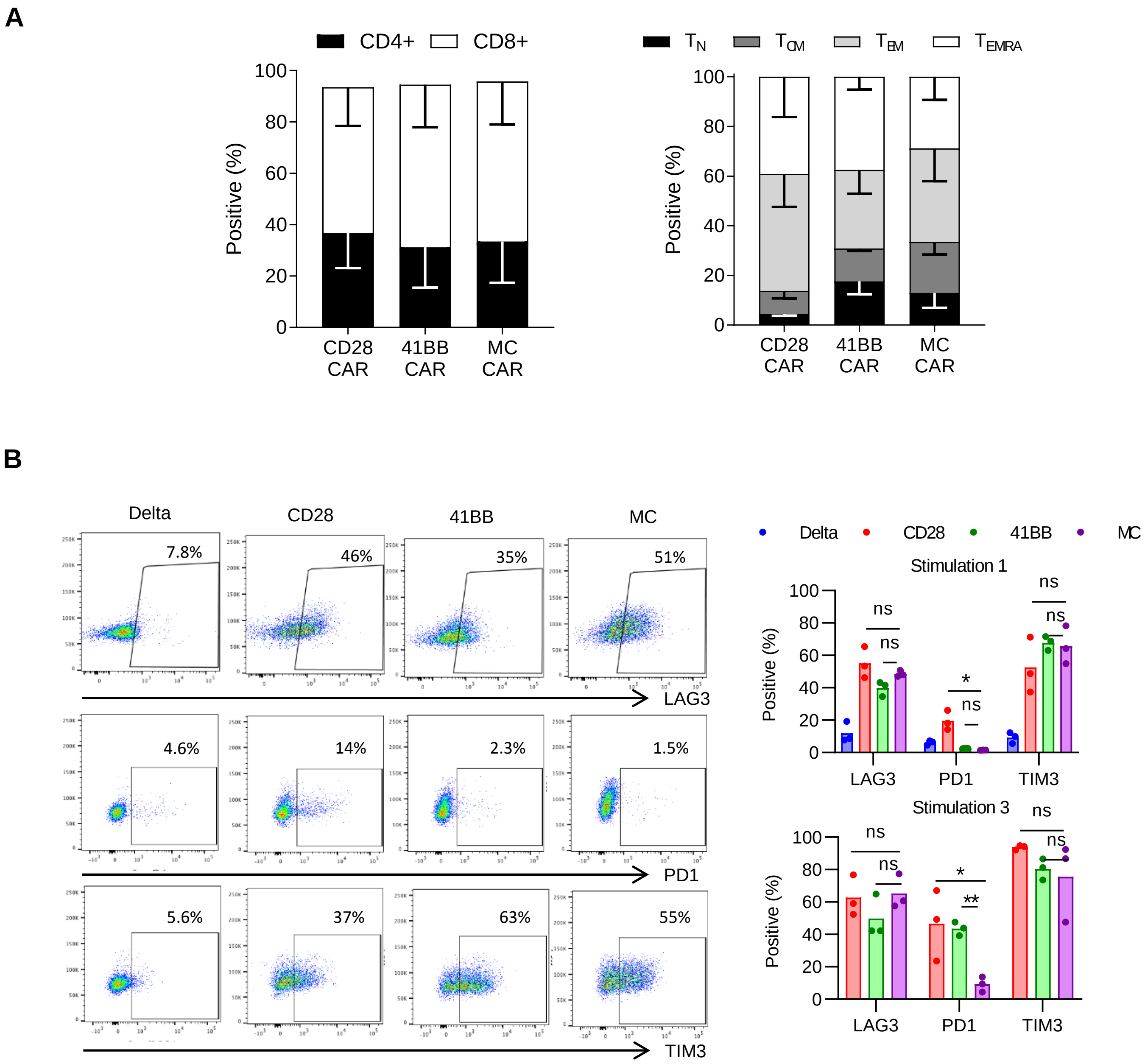

Supplemental Figure 4: CAR T cells show only minor differences in phenotype and exhaustion markers post multiple tumor stimulations. CAR T cells were co-cultured with tumor cells at a 2:1 E:T ratio with weekly restimulation using fresh tumor cells. Flow cytometry for expression of phenotypic and exhaustion markers was performed 7 days after stimulation. **A.** T cells were phenotyped as in Figure 2 after second stimulation with tumor cells with no significant differences in phenotype between CD28-, 41BB-, or MC-CAR T cells (n=3, 2-way ANOVA with Tukey's test for multiple comparisons). **B.** T cells were evaluated for their expression of the exhaustion markers LAG3, PD1, and TIM3 7 days after the 1st and 3rd stimulation with tumor cells. Representative flow cytometry plots for the first stimulation and summary data from all donors are shown (n=3, 2-way ANOVA with Tukey's test for multiple comparisons). All statistical tests were performed in comparison to MC-CAR T cells (p<0.05: *; p<0.01: **).

Supplemental Figure 4

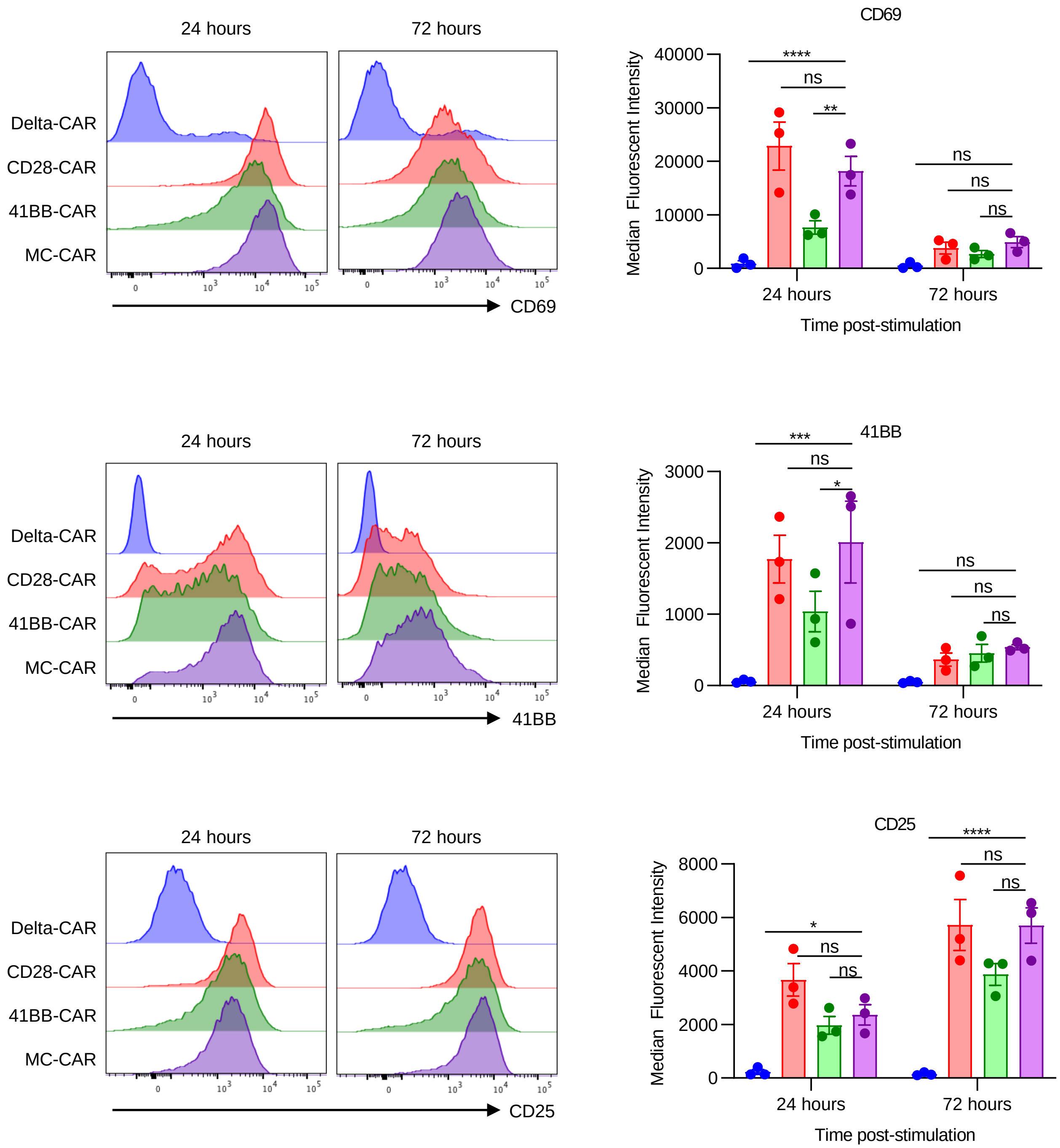

Supplemental Figure 5: Expression of activation markers on EphA2 CAR T cells. EphA2 CAR T cells were stimulated with LM7 tumor cells at a 2:1 effector:target ratio. Expression of CD69, 41BB, and CD25 was assessed by flow cytometry at 24 hours and 72 hours post-stimulation. Representative flow plots (left) and summary data (right) are shown (n=3, mean with SEM 2-way ANOVA with Dunnett's multiple comparisons test). All statistics shown are in comparison to MC-CAR T cells (not significant: ns, p<0.05: *, p<0.01: **, p<0.001: ***, p<0.0001: ****).

Supplemental Figure 5

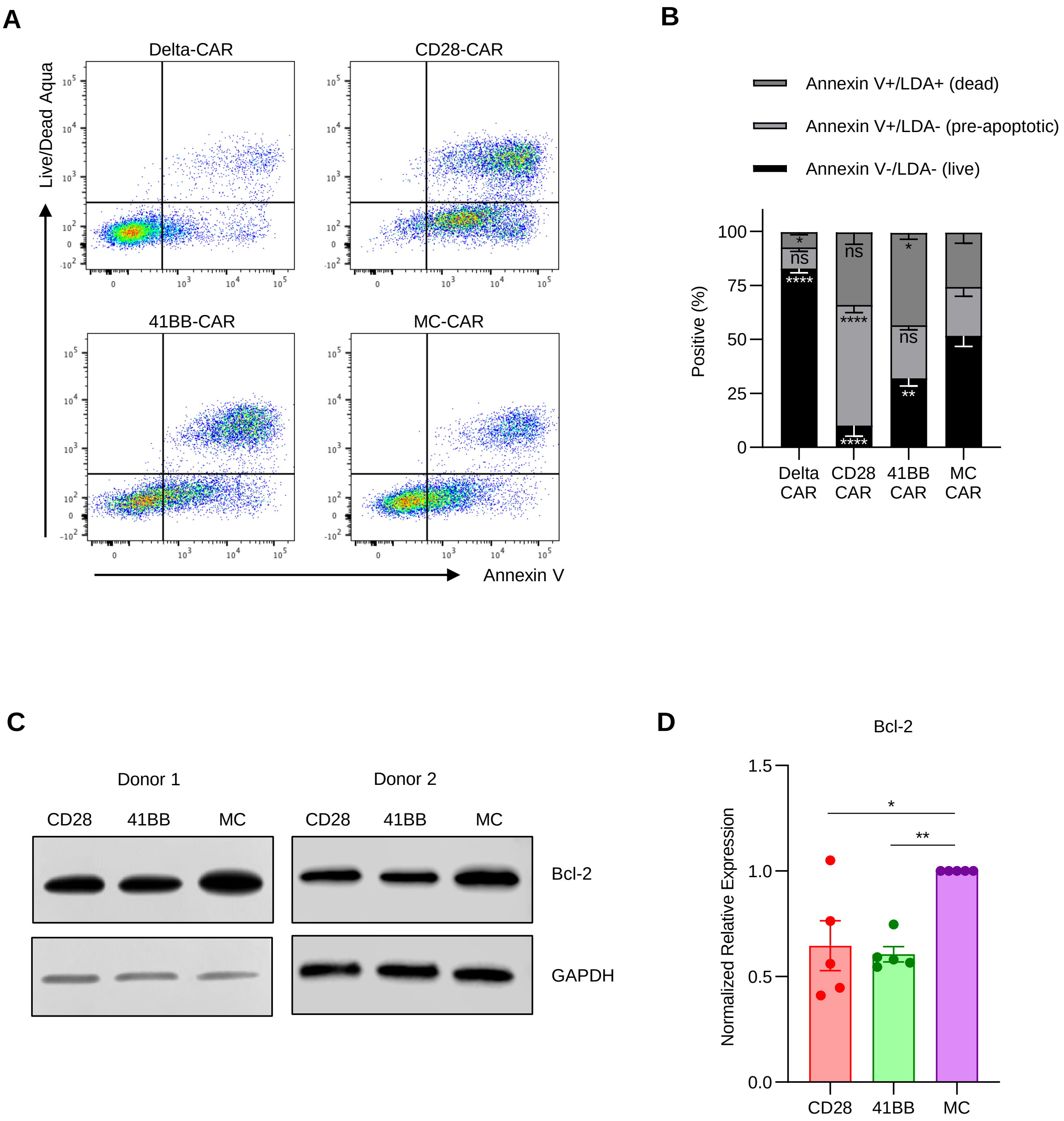

Supplemental Figure 6, A-D

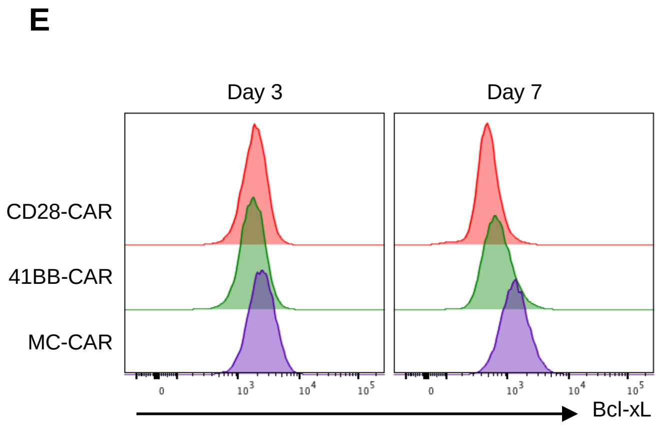

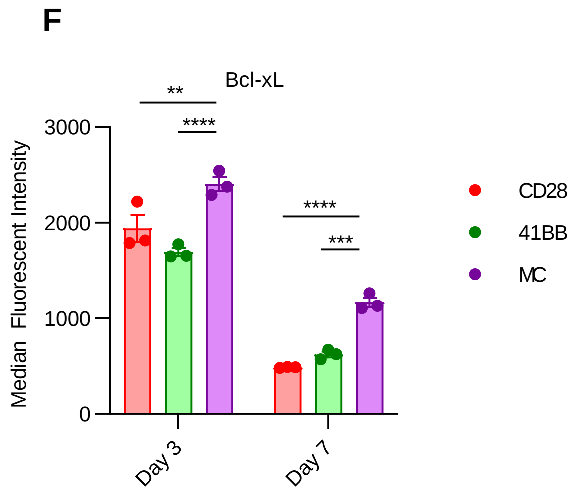

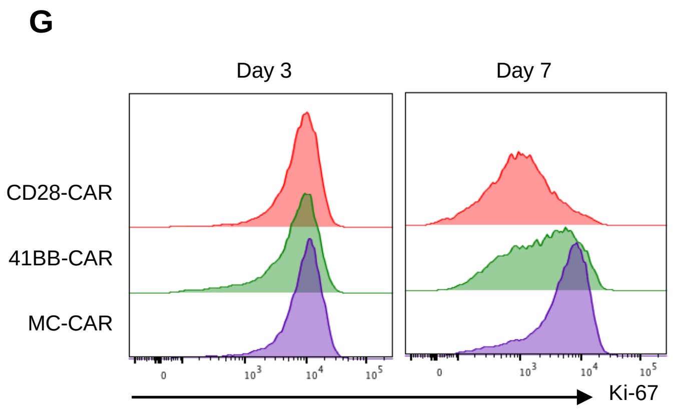

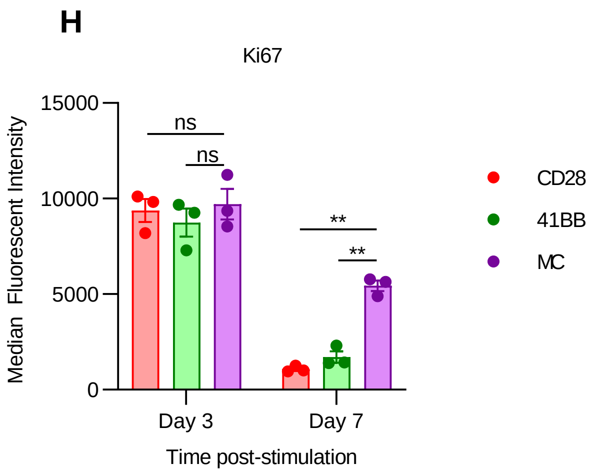

Supplemental Figure 6: MC-CAR T cells exhibit less susceptibility to AICD and remain proliferative longer than CD28- or 41BB-CAR T cells. A-B. EphA2 CAR T cells were stimulated with recombinant human EphA2 protein for 24 hours before being stained with a dead-cell dye (Live/Dead Aqua; LDA) and Annexin V. Representative flow plots **(A)** and summary data **(B)** are shown (n=4, mean with SEM, 2-way ANOVA with Tukey's test for multiple comparisons). **C-D.** Western Blot was used to determine the expression of Bcl-2 in EphA2 CAR T cells 24 hours after stimulation with recombinant EphA2 protein. GAPDH was used as a loading control. **C.** Representative blots for two donors are shown **D.** Pixel density was quantified using Li-Cor Image Studio. For each donor, expression of Bcl-2 was normalized to GAPDH and then plotted relative to expression in MC-CAR T cells (n=5, one-way ANOVA with Tukey's test for multiple comparisons). **E-H.** EphA2 CAR T cells were stimulated with LM7 tumor cells at a 2:1 effector target ratio and stained for Bcl-xL **(E,F)** and Ki-67 **(G,H)** at Day 3 and Day 7 post-stimulation. Representative flow plots **(E,G)** and summary data **(F,H)** are shown (n=3, mean with SEM, 2-way ANOVA with Tukey's test for multiple comparisons). All statistics shown are in comparison to MC-CAR T cells (not significant: ns, $p<0.05$: *, $p<0.01$: **, $p<0.001$: ***, $p<0.0001$: ****).

Supplemental Figure 6, E-H

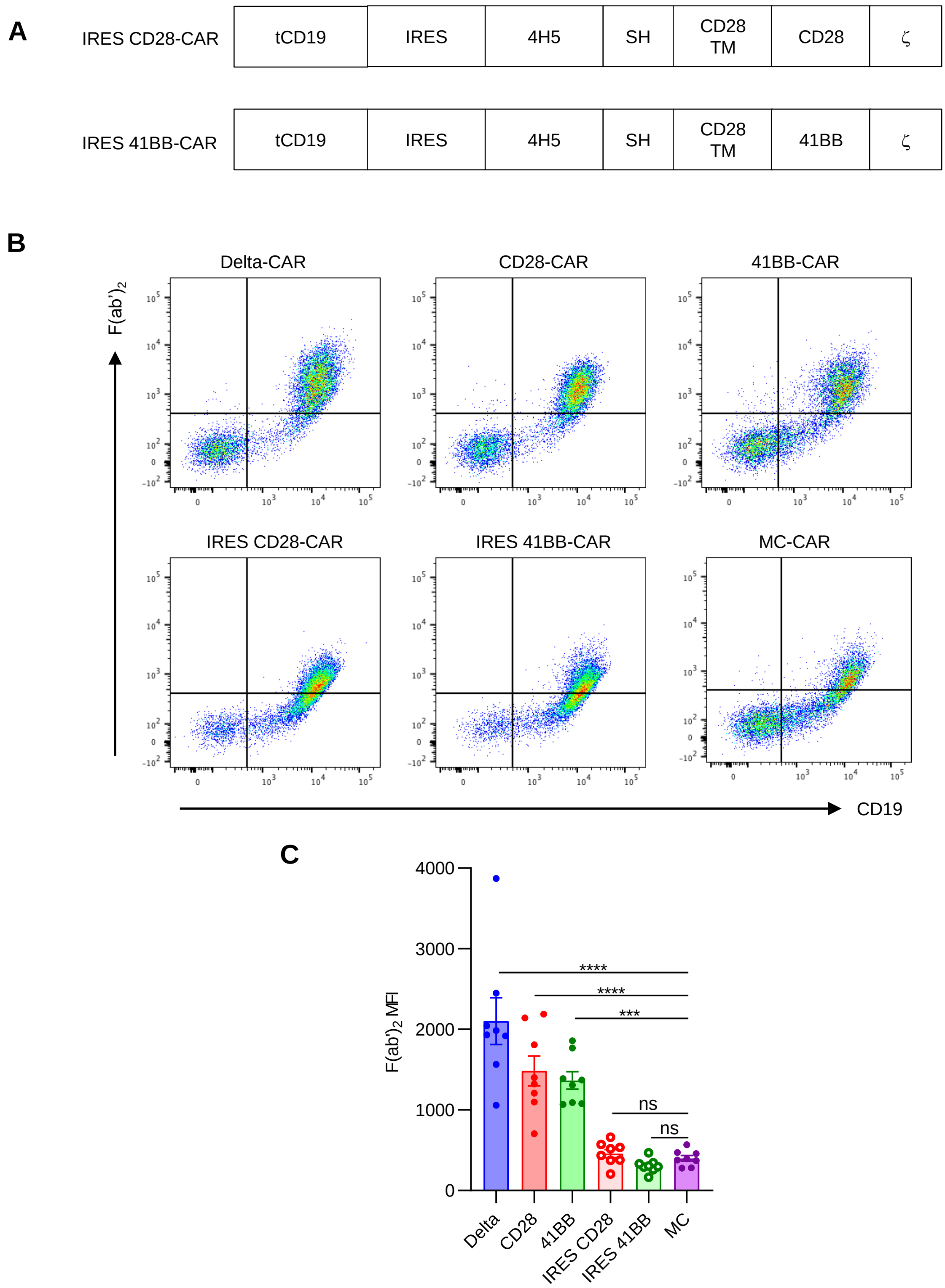

Supplemental Figure 7: Generation of low-MFI CD28- and 41BB-CAR T cells. A. Schematic of IRES CD28- and IRES 41BB-CARs generated to lower CAR expression on the cell surface. **B.** T cells were assessed for transduction (CD19) and expression of the CAR ($F(ab')_2$) at Day 7-12 post-transduction using flow cytometry. Representative flow plots are shown. **C.** The level of CAR expression at the cell surface was assessed by median fluorescent intensity (MFI) of $F(ab')_2$ in transduced (CD19+) cells (n=8, mean with SEM, one-way ANOVA with Dunnett's multiple comparison test). All statistics shown are in comparison to MC-CAR T cells (not significant: ns, $p<0.001$: ***, $p<0.0001$: ****).

Supplemental Figure 7

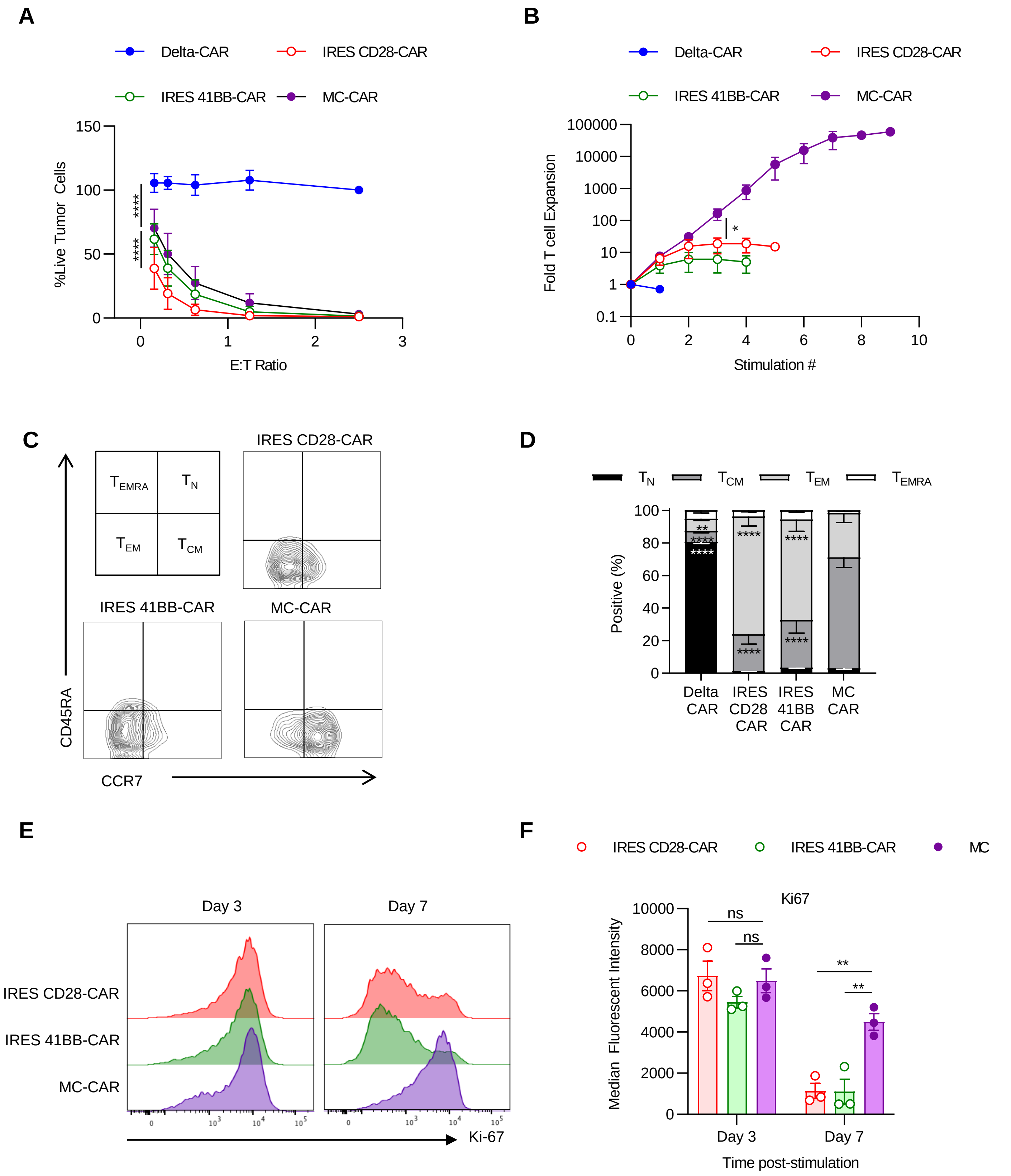

Supplemental Figure 8, A-F

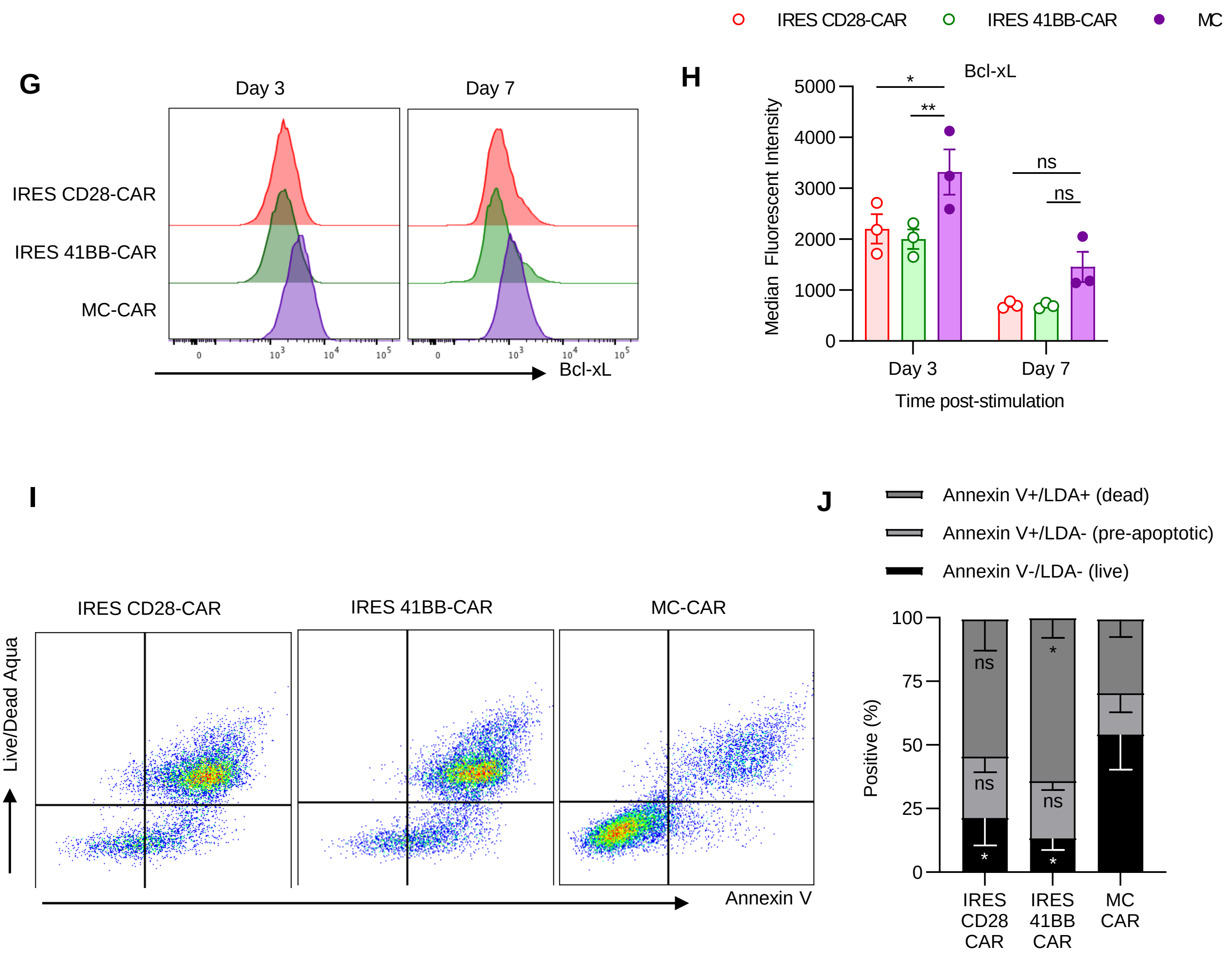

Supplemental Figure 8: MC-CAR T cells outperform low-MFI CD28- and 41BB-CAR T cells. A. EphA2 CAR T cells were co-cultured with LM7 tumor cells at varying effector:target (E:T) ratios for 24-hours before the number of live tumor cells was assessed using an MTS assay (n=5, mean with SEM, 2-way ANOVA with Dunnett's multiple comparisons test). **B.** EphA2 CAR T cells were co-cultured with LM7 tumor cells at a 2:1 effector:target ratio. One week after stimulation, T cells were counted and restimulated with fresh tumor cells at the same 2:1 E:T ratio. T cells were counted and restimulated on a weekly basis until they stopped expanding or killing tumor cells (n=4, mean with SEM, one-way ANOVA with Dunnett's multiple comparisons test at stimulation 3, stats refer to MC-CAR vs. IRES CD28-CAR and MC-CAR vs IRES 41BB-CAR). **C-D.** EphA2 CAR T cells were co-cultured with LM7 tumor cells at a 2:1 E:T ratio. At Day 7 post-stimulation, T cell phenotype was determined with flow cytometry. Representative flow plots **(C)** and summary phenotype data for all donors **(D)** are shown. Unstimulated Delta-CAR T cells were used as a control. E**-H.** EphA2 CAR T cells were stimulated with LM7 tumor cells at a 2:1 effector target ratio and stained for Ki67 **(E,F)** and Bcl-xL **(G,H)** at Day 3 and Day 7 post-stimulation. Representative flow plots **(E,G)** and summary data **(F,H)** are shown (n=3, mean with SEM, 2-way ANOVA with Tukey's multiple comparisons test). **I-J.** At day 7 post-stimulation with LM7 tumor cells, EphA2 CAR T cells were stained with a dead-cell dye (Live/Dead Aqua; LDA) and Annexin V. Representative flow plots **(I)** and summary data **(J)** are shown (n=3, mean with SEM, 2-way ANOVA with Tukey's multiple comparisons test). All statistics shown are in comparison to MC-CAR T cells (not significant: ns, p<0.05: *, p<0.01: **, p<0.001: ***, p<0.0001: ****).

Supplemental Figure 8, G-J

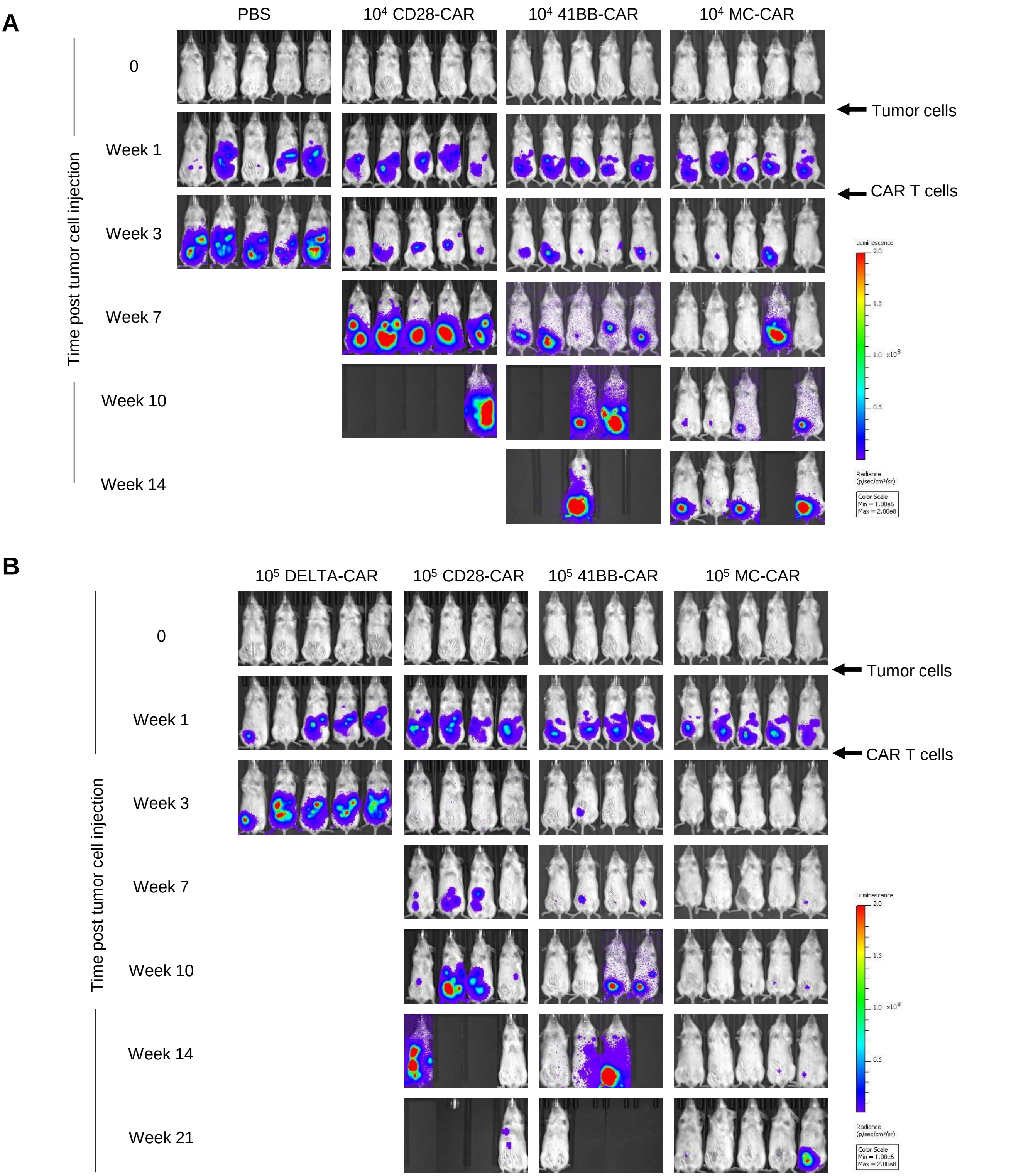

Supplemental Figure 9: MC-CAR T cells exhibit superior anti-tumor activity in LM7 model. Related to Figure 3. NSG mice were injected with $1x10^6$ LM7-ffLuc i.p. One week later they were injected i.p. with $1x10^4$ **(A)** or $1x10^5$ **(B)** CD28-, 41BB-, or MC-CAR T cells. PBS **(A)** or $1x10^5$ **(B)** Delta-CAR T cells were used as controls. Representative bioluminescence images are shown. Experiments were repeated twice with CAR T cells generated from two different healthy donors.

Supplemental Figure 9

A

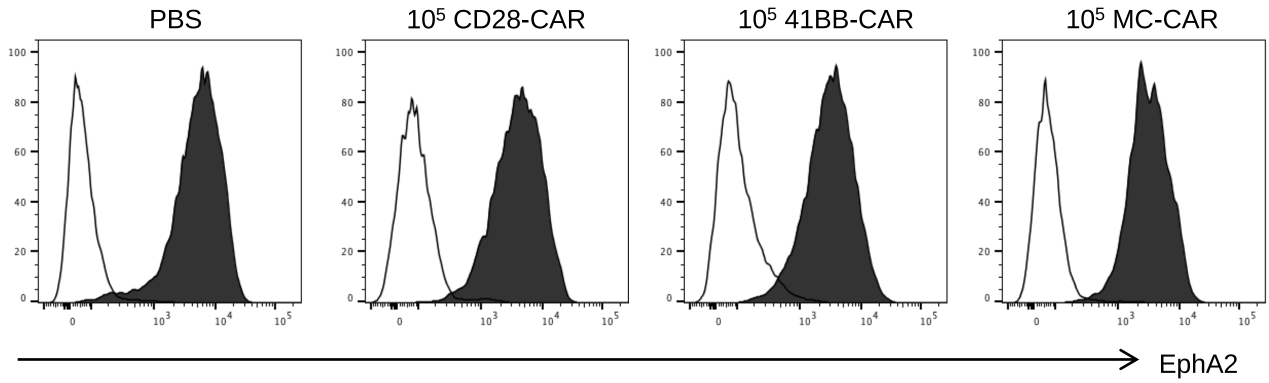

B

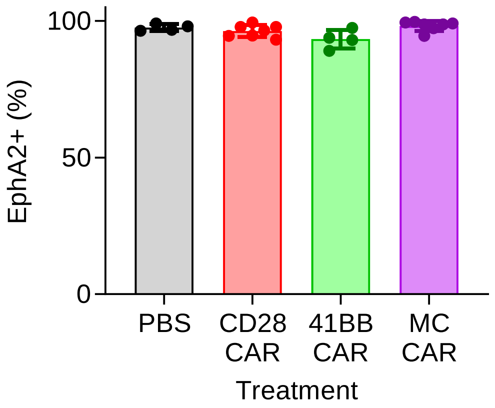

Supplemental Figure 10: Recurrent LM7 tumors continue to express the targeted antigen. Tumors were removed from mice after they reached our BLI-defined endpoint. Tumors were dissociated with the GentleMACS dissociation kit, washed, and stained for EphA2. **A.** Representative flow cytometry plots of isotype controls (white histograms) and EphA2 expression (black histograms) on tumors that were removed from mice treated with PBS or 10^5 CD28-, 41BB-, or MC-CAR T cells. **B.** Summary plot of EphA2 expression on recurrent tumors in mice treated with PBS or EphA2 CAR T cells (n=4-7 per construct, both CAR T cell doses, 10^4 and 10^5 included).

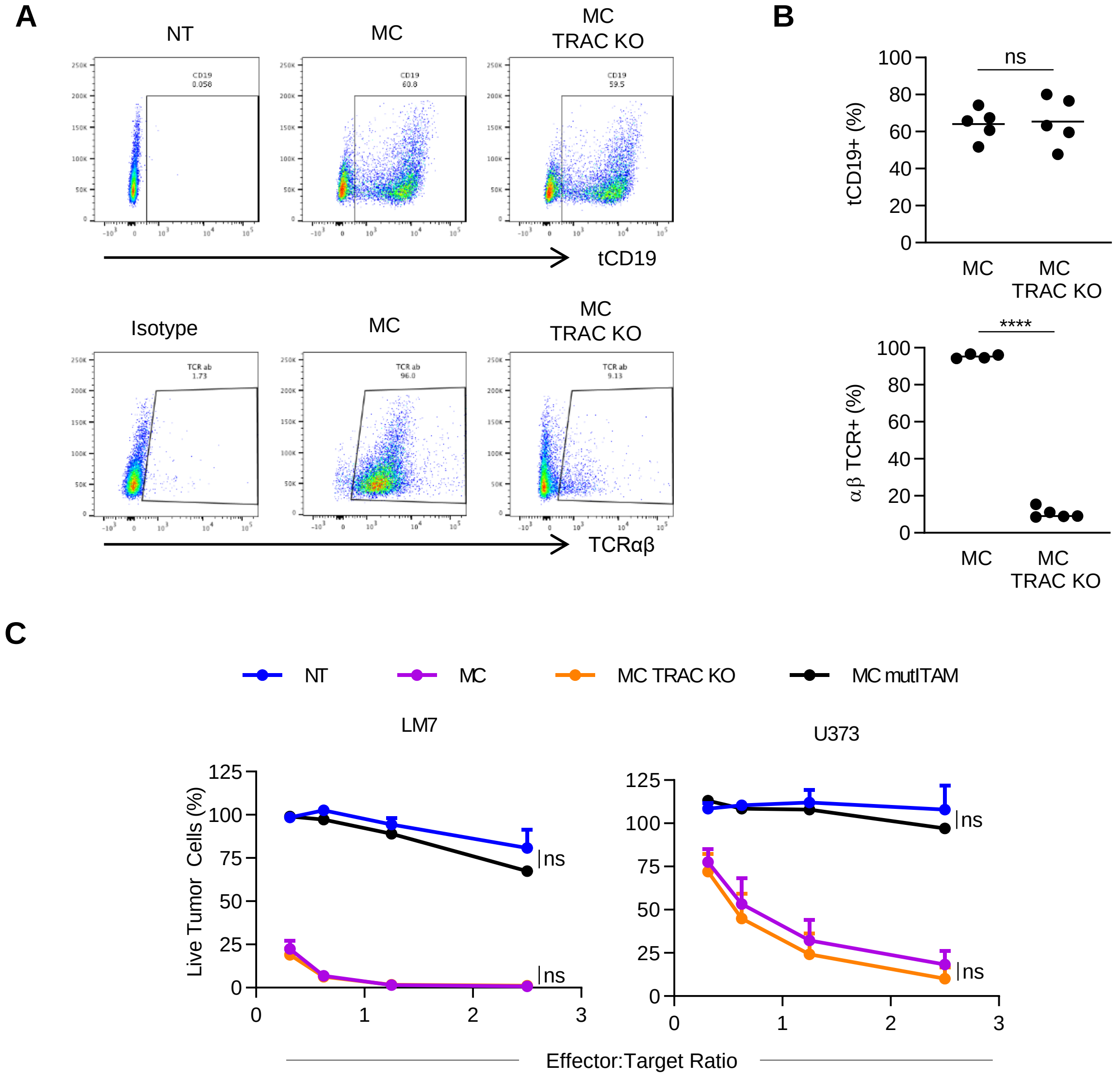

Supplemental Figure 11A-C

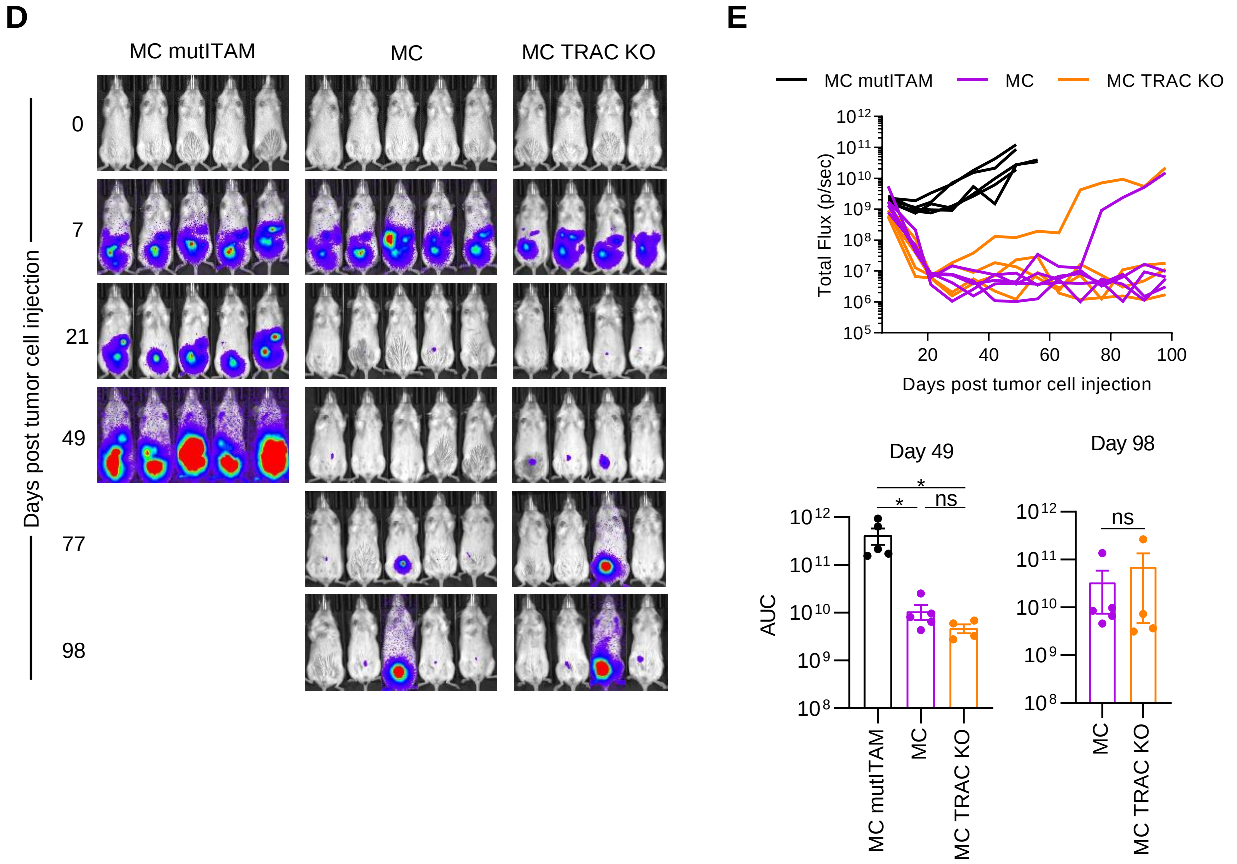

Supplemental Figure 11: Antitumor activity of MC-CAR T cells depends on ζ signaling domain and is independent of endogenous αβ TCR expression. A. MC TRAC KO-CAR T cells were generated using CRISPR-Cas9. Representative flow cytometry plots for CD19 and αβTCR expression on MC-CAR, MC TRAC KO-CAR T cells and non-transduced (NT) T cells. **B.** Summary plots for tCD19 and αβTCR expression for all donors used to compare MC and MC TRAC KO (n=4-5, unpaired t-test, ns=not significant, p<0.0001:****). **C.** 24-hour MTS assay using LM7 and U373 as targets and as effectors MC TRAC KO-CAR T cells, MC-CAR T cells, NT T cells, or T cells expressing a MC-CAR with mutated ITAMs (MC mutITAM-CAR T cells) (n=2-3; two-way ANOVA with Tukey's test for multiple comparisons). **D-E.** NSG mice were injected i.p. with 1x10^6 LM7-ffLuc tumor cells. One week later, they were injected with 1x10^5 MC mutITAM-CAR, MC-CAR, or MC TRAC KO-CAR T cells (n=4-5 per group). **D.** Representative bioluminescent images. **E.** Total flux from tumor cells in all mice and area under the curve analysis at day 49 and day 98 post tumor cell injection.

Supplemental Figure 11D,E

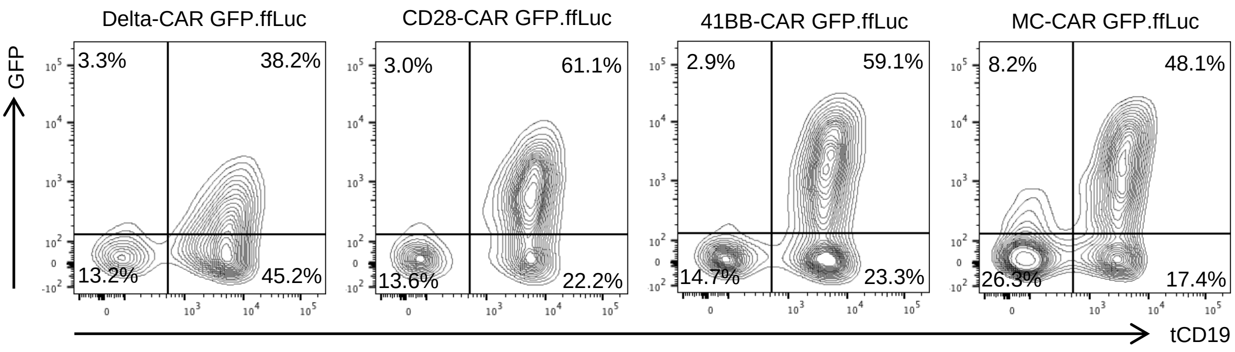

Supplemental Figure 12: Generation of CAR T cells expressing GFP.ffLuc. CAR T cells were transduced with retroviral vectors encoding the respective CAR (tCD19+) and GFP.ffLuc. Transduction efficiency was evaluated using flow cytometry.

Supplemental Figure 12

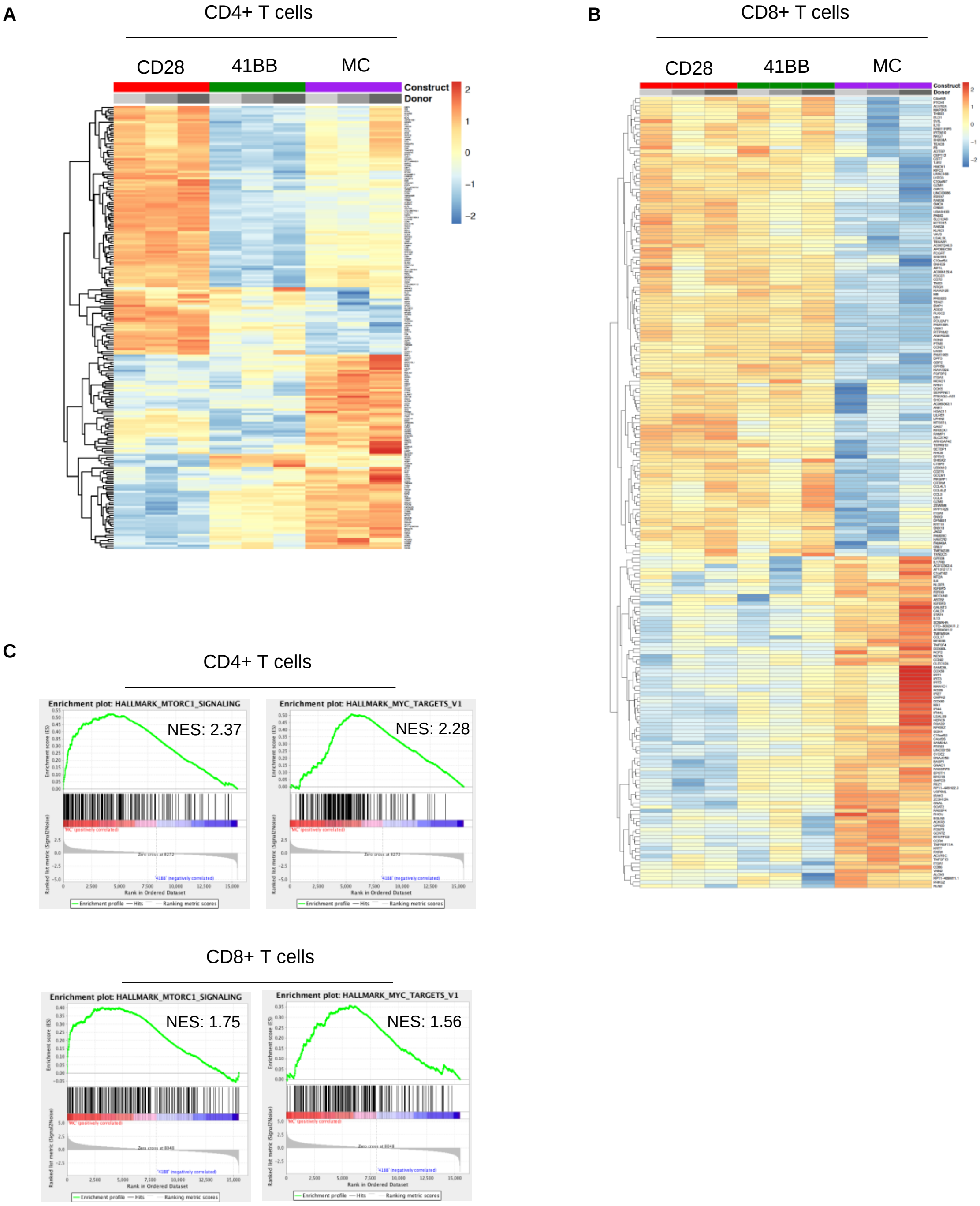

Supplemental Figure 13: MC-CAR T cells express a distinct transcriptional profile post-activation. CAR T cells from 3 healthy donors were stimulated with LM7 tumor cells and sorted after 24 hours into CD4+CAR+ and CD8+CAR+ subsets. RNA was extracted from sorted cells and sequenced on an Illumina NovaSeq platform. **A.** Top differentially expressed genes in CD4+ CAR T cells. **B.** Top differentially expressed genes in CD8+ CAR T cells. **C.** Gene Set Enrichment Analysis (GSEA) of MC- vs 41BB-CAR T cells was performed after removing baseline expression differences between the two constructs. Normalized enrichment scores (NES) are shown.

Supplemental Figure 13

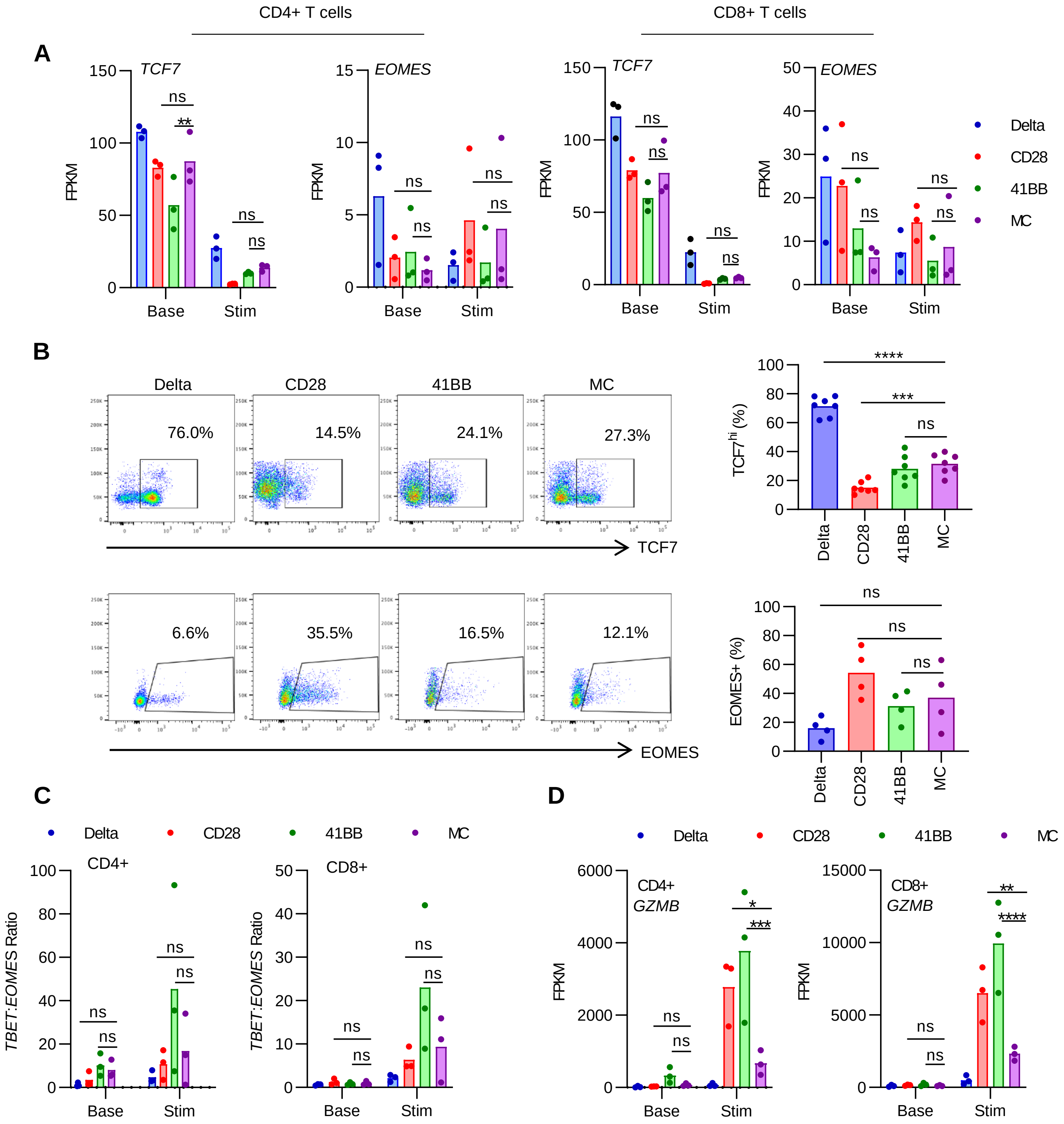

Supplemental Figure 14: CD28- and 41BB-CAR T cells display a more differentiated phenotype as judged by Granzyme B expression. A. Expression of *TCF7* and *EOMES* at baseline and after 24 hour stimulation with LM7 in CD4+ (left) and CD8+ (right) T cells (n=3, two-way ANOVA with Tukey's test for multiple comparisons). **B.** CAR T cells were stimulated with recombinant human EphA2 protein for 24 hours before intracellular staining for the transcription factors TCF7 and EOMES. Representative flow plots and summary of expression for all donors are shown (TCF7: n=7, EOMES: n=4, one-way ANOVA with Tukey's test for multiple comparisons). C. The ratio of *TBET:EOMES* expression in CD4+ (left) and CD8+ (right) T cells at baseline and after 24 hour stimulation with LM7 (n=3, two-way ANOVA with Tukey's test for multiple comparisons). **D.** Expression of *GZMB* (encodes Granzyme B) at baseline and after 24 hour stimulation with LM7 in CD4+ (left) and CD8+ (right) T cells (n=3, two-way ANOVA with Tukey's test for multiple comparisons. All statistical tests were performed in comparison to MC-CAR T cells (p<0.05: *; p<0.01: **, p<0.001: ***, p<0.0001: ****).

Supplemental Figure 14

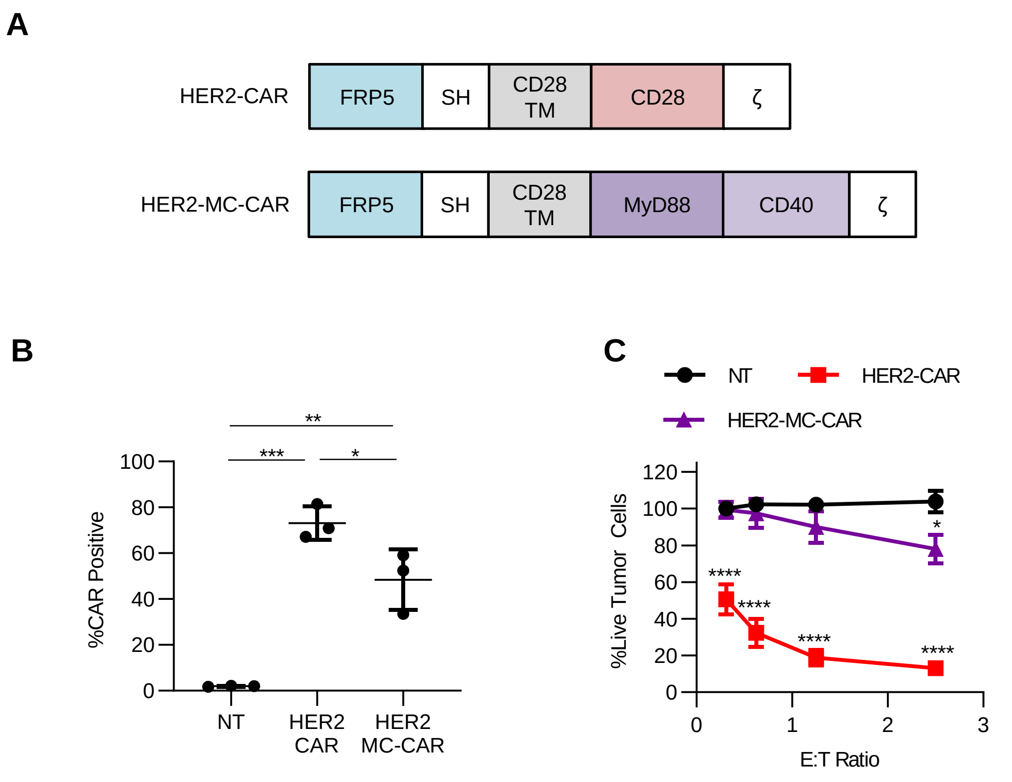

Supplemental Figure 15: T cells expressing HER2-CARs with MC endodomain have minimal cytolytic activity. A. Scheme of HER2-CAR constructs. **B.** T cells were stained with anti-mouse IgG, F(ab')$_2$ fragment specific-AF647 to detect CAR expression (n=3, one-way ANOVA with Tukey's test for multiple comparisons) **C.** An MTS assay was used to assess CAR T cell cytotoxicity against the HER2-positive cell line U373 (n=3, two-way ANOVA with Tukey's test for multiple comparisons, asterisks denote statistical analysis compared to NT; p<0.05: *, p<0.01: **, p<0.001: ***, p<0.0001: ****).

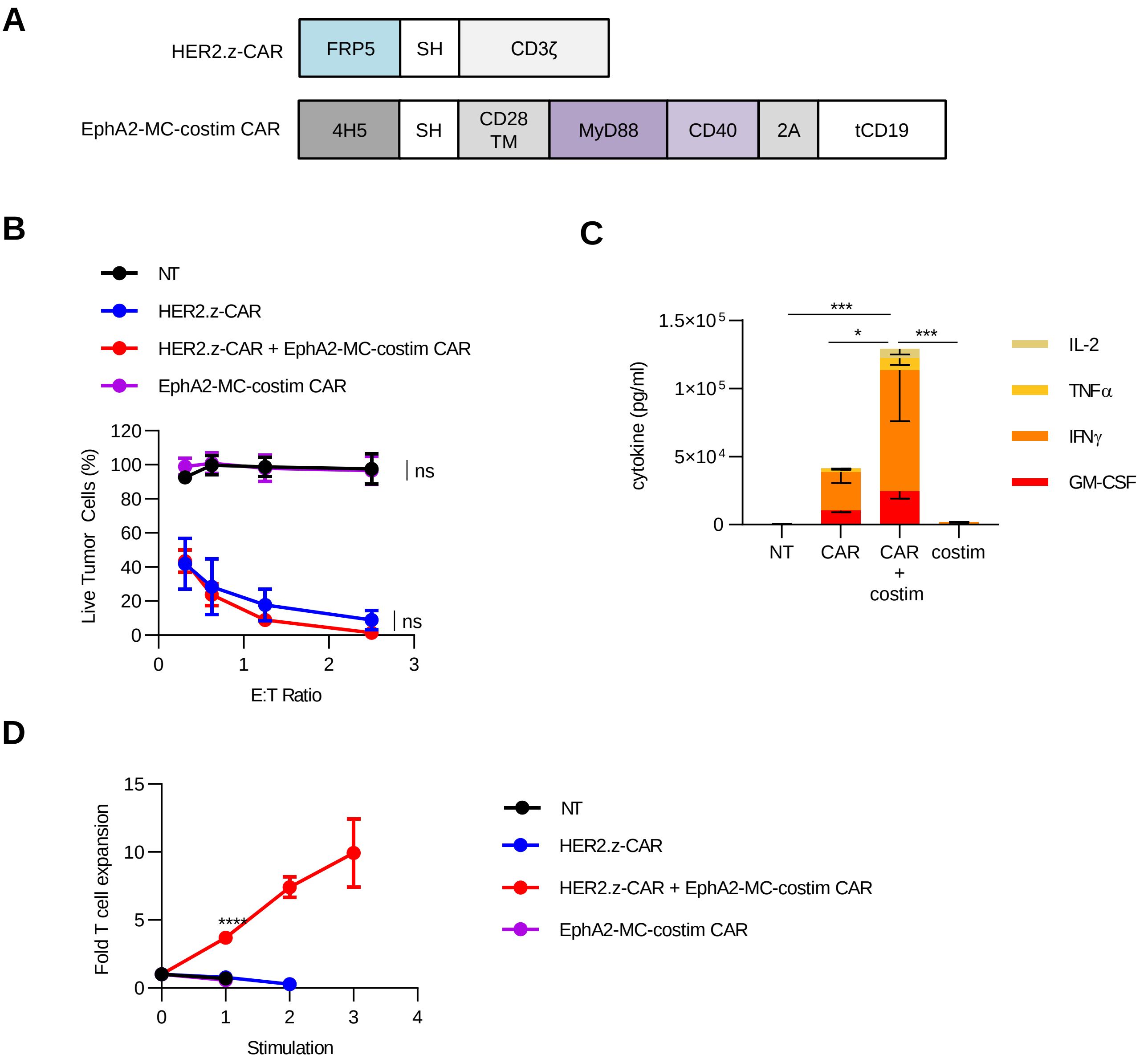

Supplemental Figure 16: MC costimulation through a 2nd CAR. A. Scheme of HER2.z-CAR and EphA2-specific MC-costimulatory CAR (EphA2-MC-costim CAR). **B.** T cells expressing HER2.z-CAR and/or MC-costim CAR were generated by retroviral transduction. 24-hour MTS assay (n=3, mean+SEM, two-way ANOVA with Tukey's test for multiple comparisons, ns=not significant). **C.** Summary of Th1 cytokine production after 24 hour CAR T cell incubation with U373 (n=3, mean + SEM, two-way ANOVA with Tukey's multiple comparisons test). **D.** T cells were co-cultured with tumor cells at a 2:1 E:T ratio with weekly restimulation against fresh tumor cells until they lost their effector function and no longer killed all the tumor cells (n=3, mean+SEM, one-way ANOVA at 1st stimulation with Tukey's test for multiple comparisons; $p<0.05$:*, $p<0.01$:**, $p<0.001$:***, $p<0.0001$:****).

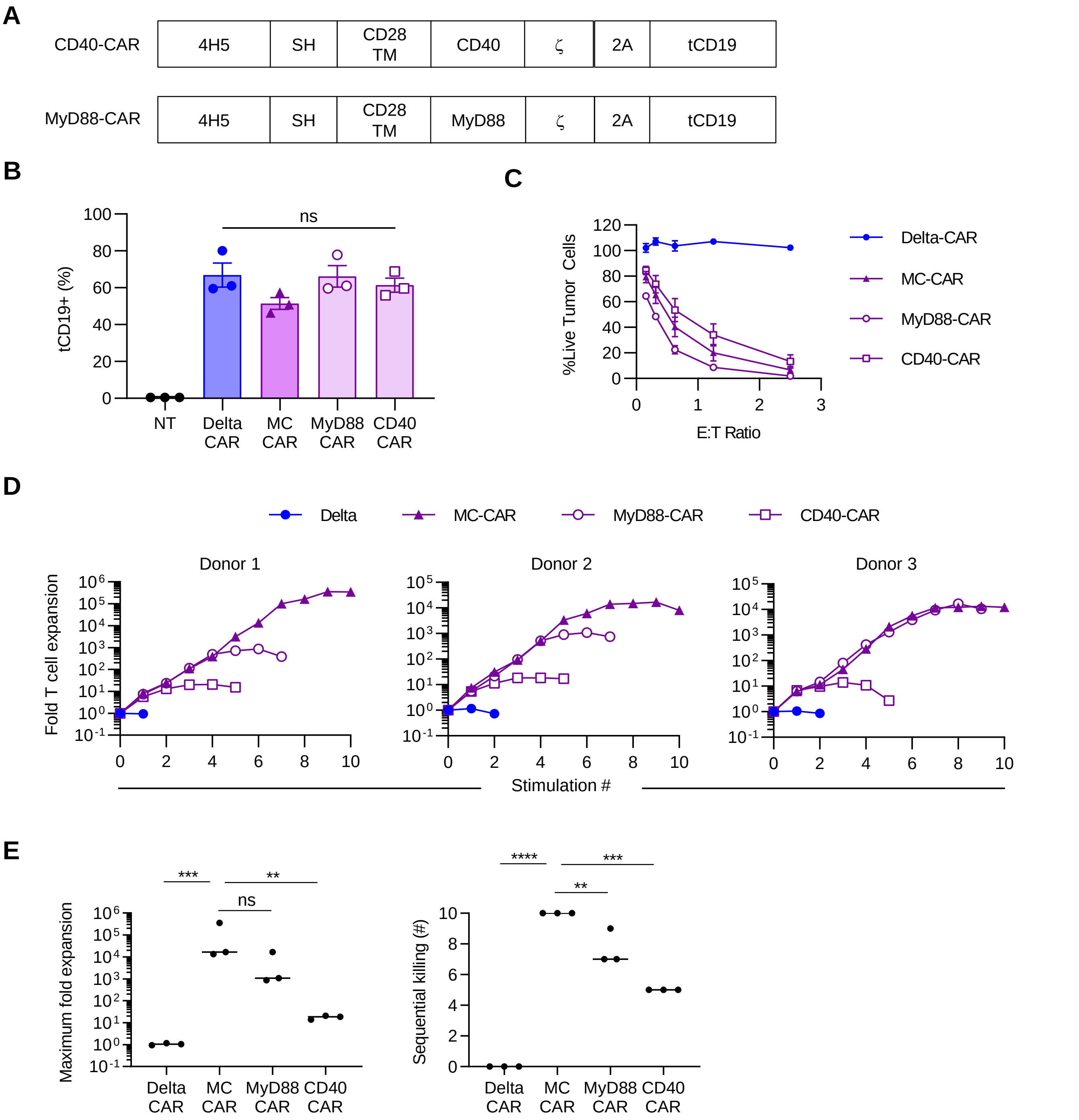

Supplemental Figure 17: *in vitro* evaluation of MyD88- and CD40-CAR T cells. A. Schematic of EphA2 MyD88 and EphA2 CD40 CAR constructs. **B.** T cells were assessed for transduction (tCD19+) at Day 8 post-transduction using flow cytometry (n=3, mean with SEM, one-way ANOVA with Dunnett's multiple comparisons test). **C.** CAR T cells were co-cultured with LM7 tumor cells at varying effector:target (E:T) ratios for 24-hours before the number of live tumor cells was assessed using an MTS assay (n=3, mean with SEM). **D-E.** CAR T cells were co-cultured with LM7 tumor cells at a 2:1 effector:target ratio. One week after stimulation, T cells were counted and restimulated with fresh tumor cells at the same 2:1 E:T ratio. T cells were counted and restimulated on a weekly basis until they stopped expanding or killing tumor cells. **D.** Each donor is shown individually. **E.** Summary of the max expansion CAR T cells from individual donors achieved and the max number of times CAR T cells were able to kill fresh tumor cells (n=3, median shown, 2-way ANOVA with Dunnett's multiple comparisons test). All statistics shown are in comparison to MC-CAR T cells (ns: not significant, p<0.01:**, p<0.001:***, p<0.0001:****).

Supplemental Figure 17
